# Supplementary figures and images for: Supplemental Oxygen Alters the Airway Microbiome in Cystic Fibrosis
Source: mSystems. 2022 Aug 24;7(5):e00364-22. doi: 10.1128/msystems.00364-22 (PMC9601246; doi:10.1128/msystems.00364-22)

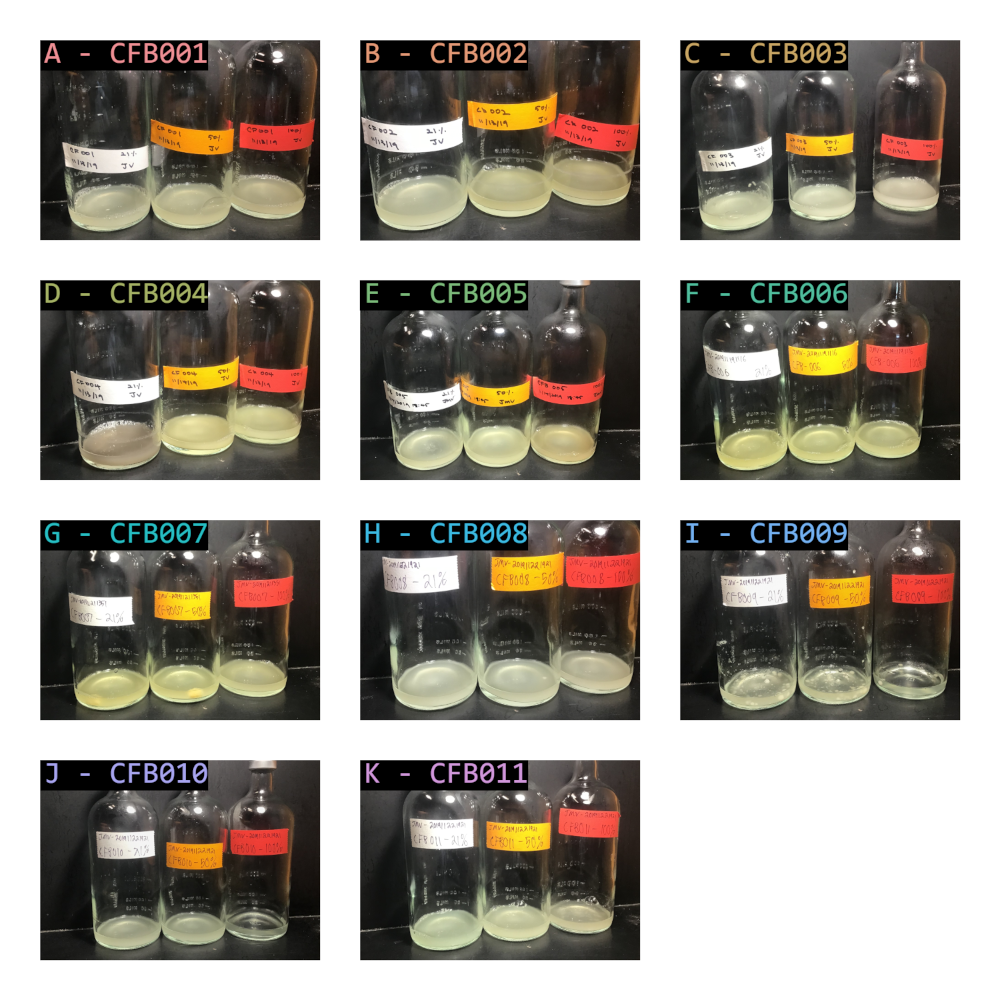

Supplement: FIG S1 [file msystems.00364-22-s0006.tif]

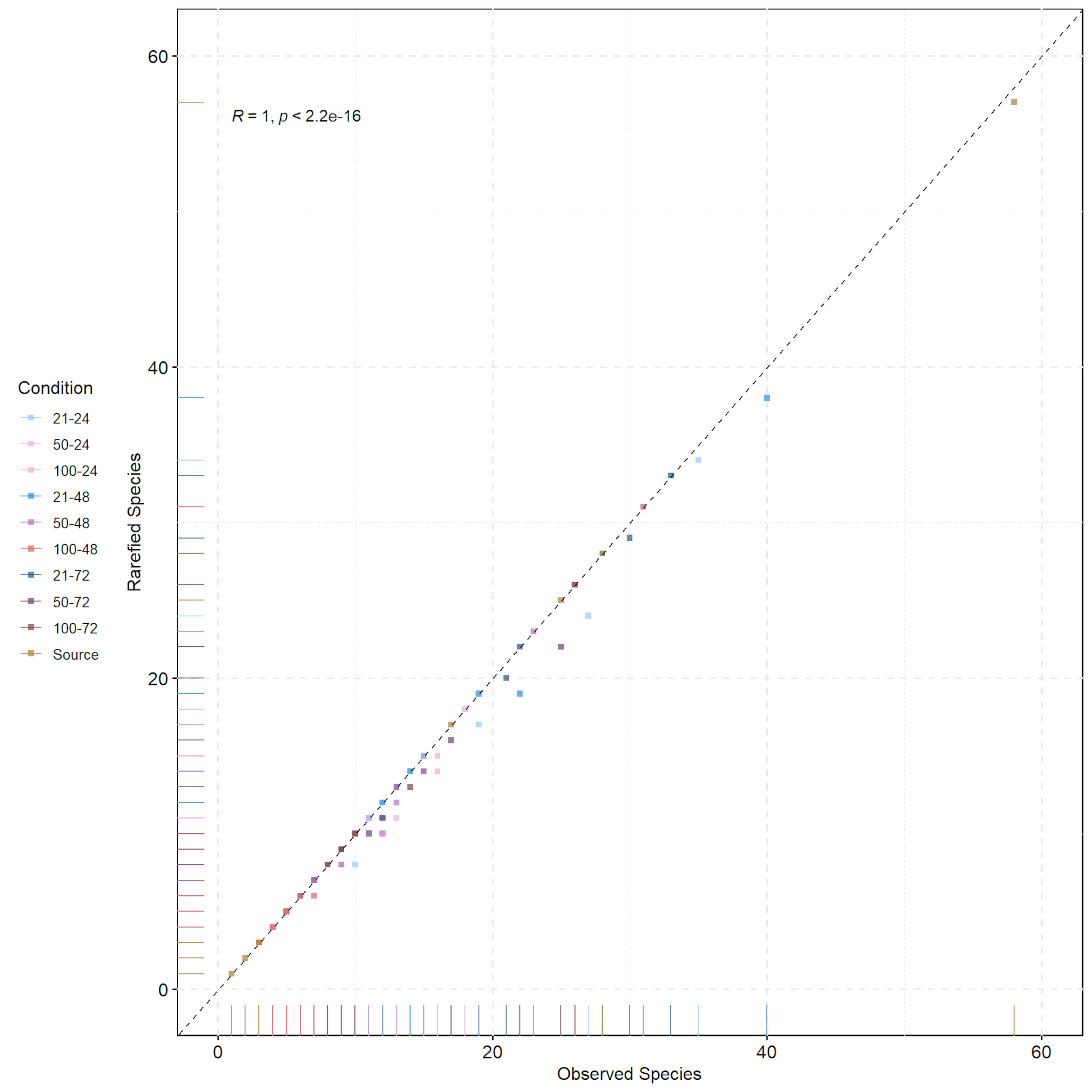

Supplement: FIG S2 [file msystems.00364-22-s0007.tif]

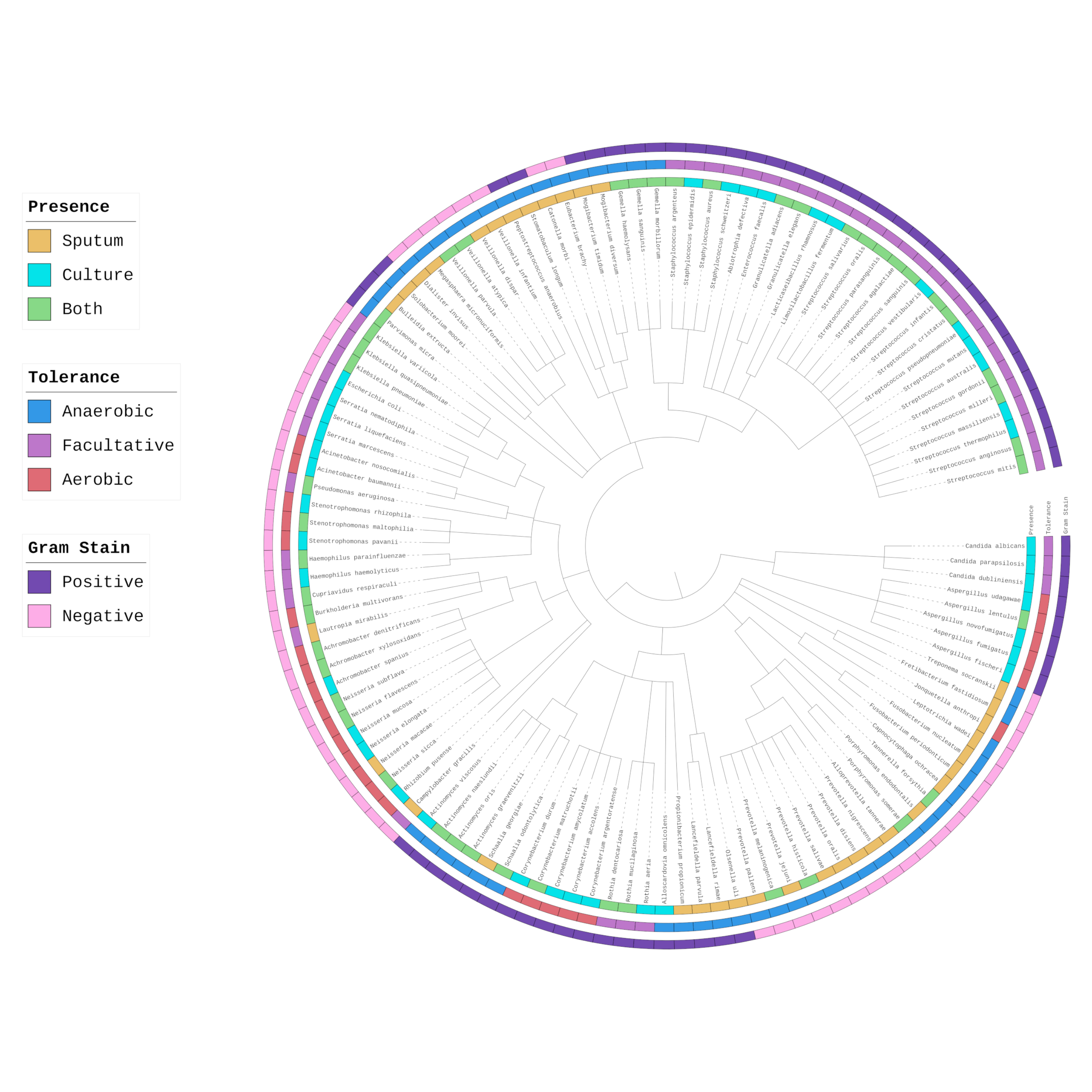

Supplement: FIG S3 [file msystems.00364-22-s0008.tif]

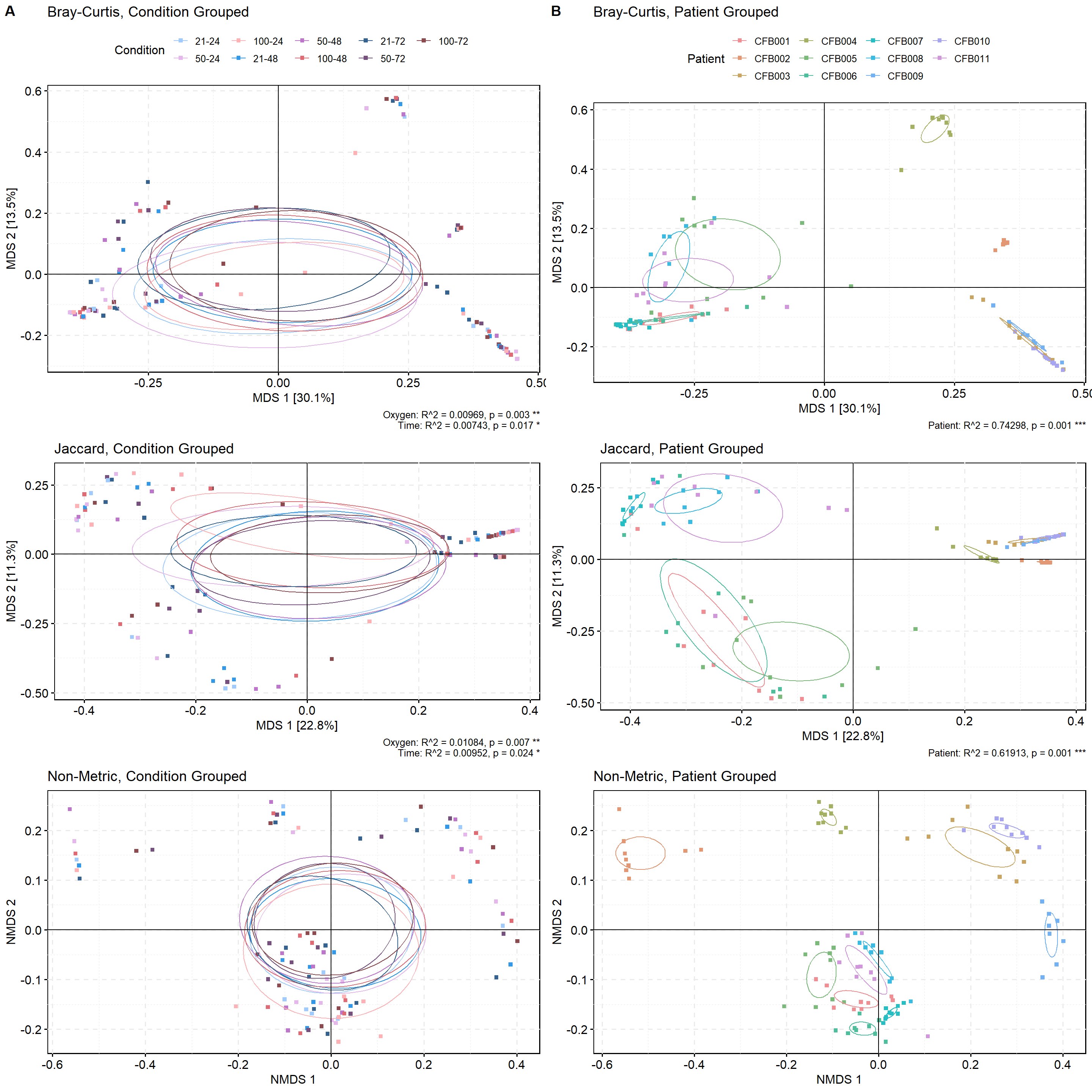

Supplement: FIG S4 [file msystems.00364-22-s0009.tif]

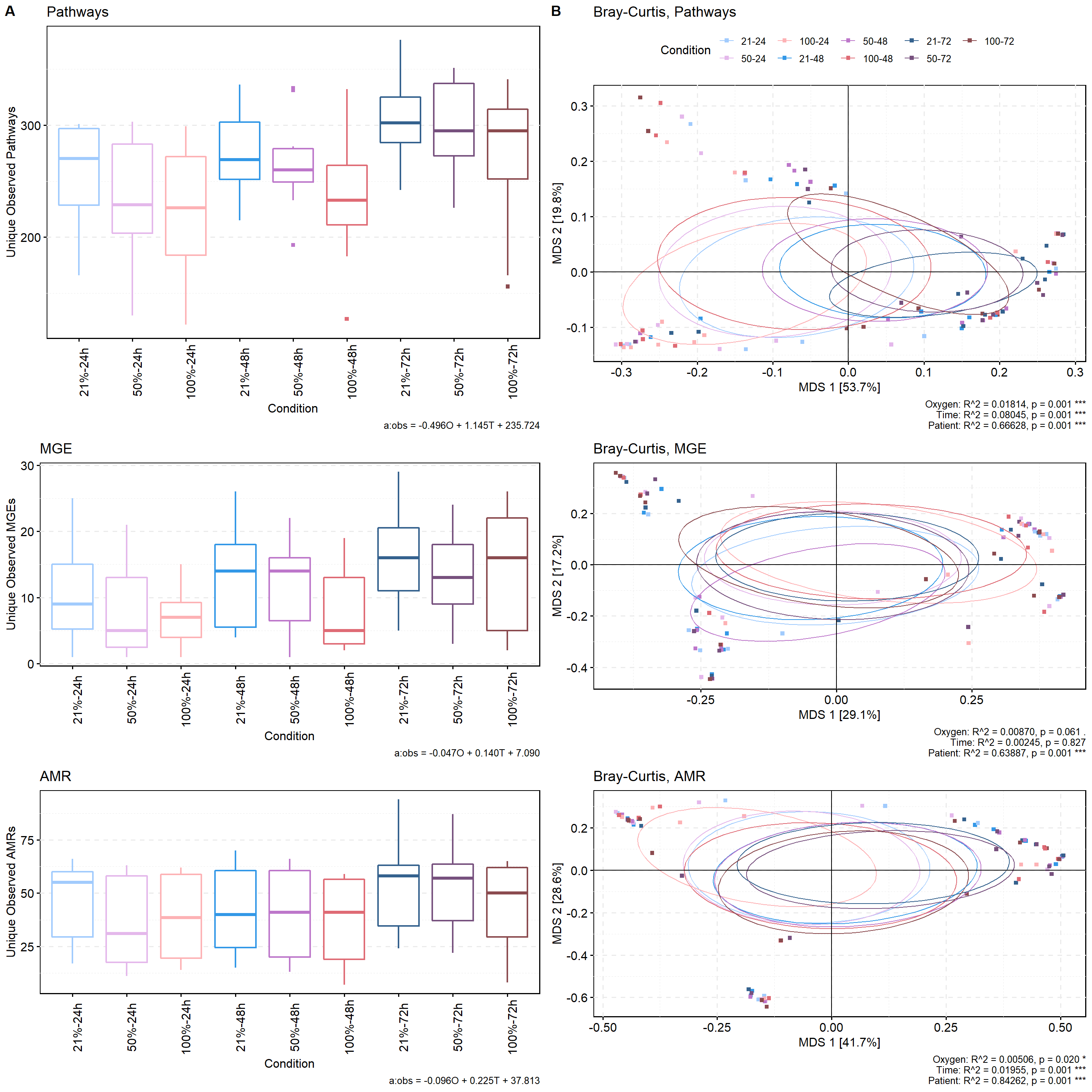

Supplement: FIG S5 [file msystems.00364-22-s0010.tif]
