# Supplementary material for: Supplemental Oxygen Alters the Airway Microbiome in Cystic Fibrosis
Source: mSystems. 2022 Aug 24;7(5):e00364-22. doi: 10.1128/msystems.00364-22 (PMC9601246; doi:10.1128/msystems.00364-22)
Supplement: TABLE S1 [file msystems.00364-22-s0001.pdf]

Microbe	Coeff	StdErr	p	q	N>0
Streptococcus sanguinis	-0.016	0.003	0.000	0.001	42
Rothia mucilaginosa	-0.019	0.005	0.000	0.012	43
Streptococcus gordonii	-0.011	0.003	0.002	0.030	48
Streptococcus mitis	-0.014	0.004	0.002	0.030	44
Actinomyces oris	-0.008	0.002	0.002	0.030	14
Streptococcus parasanguinis	-0.013	0.005	0.008	0.067	61
Candida albicans	-0.005	0.002	0.019	0.136	12
Aspergillus fumigatus	-0.007	0.003	0.023	0.155	12
Streptococcus vestibularis	-0.004	0.002	0.044	0.251	18
Schaalia odontolytica	-0.003	0.001	0.046	0.251	15
Streptococcus salivarius	-0.009	0.005	0.112	0.484	68
Rothia dentocariosa	-0.006	0.004	0.124	0.484	32
Streptococcus oralis	-0.007	0.005	0.125	0.484	56
Granulicatella elegans	-0.006	0.004	0.168	0.595	37
Streptococcus anginosus	-0.006	0.004	0.177	0.595	45
Granulicatella adiacens	-0.006	0.005	0.181	0.595	43
Streptococcus cristatus	-0.003	0.003	0.216	0.595	11
Streptococcus agalactiae	+0.005	0.005	0.339	0.740	14
Burkholderia multivorans	-0.002	0.003	0.420	0.841	15
Gemella sanguinis	-0.003	0.003	0.443	0.859	17
Pseudomonas aeruginosa	-0.006	0.008	0.468	0.859	50
Streptococcus infantis	-0.001	0.003	0.606	0.960	24
Staphylococcus aureus	-0.002	0.009	0.829	0.993	60
Abiotrophia defectiva	-0.000	0.001	0.849	0.993	10
Stenotrophomonas maltophilia	-0.001	0.004	0.862	0.993	18
Serratia marcescens	+0.000	0.003	0.882	0.993	12
Staphylococcus argenteus	-0.000	0.005	0.929	0.993	55
Klebsiella pneumoniae	+0.000	0.007	0.955	0.993	17
Klebsiella quasipneumoniae	+0.000	0.004	0.973	0.993	14
Klebsiella variicola	+0.000	0.005	0.992	0.993	15
