# Supplementary material for: Supplemental Oxygen Alters the Airway Microbiome in Cystic Fibrosis
Source: mSystems. 2022 Aug 24;7(5):e00364-22. doi: 10.1128/msystems.00364-22 (PMC9601246; doi:10.1128/msystems.00364-22)
Supplement: TABLE S2 [file msystems.00364-22-s0002.pdf]

Pathway	Coeff	StdErr	p	q	N > 0
PWY.5030.L.histidine.degradation.III	-0.063	0.011	8.32E-08	5.45E-06	63
P185.PWY.formaldehyde.assimilation.III..dihydroxyacetone.cycle.	-0.050	0.009	1.85E-07	9.07E-06	64
PWY.5345.superpathway.of.L.methionine.biosynthesis..by.sulfhydrylation.	-0.041	0.007	3.84E-07	1.12E-05	78
PYRIDNUCSAL.PWY.NAD.salvage.pathway.I	-0.039	0.007	9.78E-07	1.52E-05	66
PWY.3781.aerobic.respiration.I..cytochrome.c.	-0.043	0.008	1.04E-06	1.54E-05	90
GLYCOCAT.PWY.glycogen.degradation.I..bacterial.	-0.044	0.009	1.40E-06	1.75E-05	84
PWY.7198.pyrimidine.deoxyribonucleotides.de.novo.biosynthesis.IV	-0.048	0.009	1.64E-06	1.96E-05	77
PWY.7279.aerobic.respiration.II..cytochrome.c...yeast.	-0.044	0.009	1.99E-06	2.19E-05	87
PWY66.389.phytol.degradation	-0.030	0.006	2.46E-06	2.51E-05	96
PWY66.388.fatty.acid.alpha.oxidation.III	-0.039	0.008	3.00E-06	2.91E-05	59
PWY.7328.superpathway.of.UDP.glucose.derived.O.antigen.building.blocks.biosynthe	-0.064	0.013	4.21E-06	3.72E-05	62
PWY.5920.superpathway.of.heme.biosynthesis.from.glycine	-0.045	0.009	4.46E-06	3.85E-05	87
PWY.6527.stachyose.degradation	-0.041	0.008	4.97E-06	4.16E-05	77
PWY.5747.2.methylcitrate.cycle.II	-0.034	0.007	6.64E-06	5.16E-05	60
MET.SAM.PWY.superpathway.of.S.adenosyl.L.methionine.biosynthesis	-0.019	0.004	7.12E-06	5.49E-05	98
HOMOSER.METSYN.PWY.L.methionine.biosynthesis.I	-0.020	0.004	7.89E-06	5.79E-05	98
PWY3O.19.ubiquinol.6.biosynthesis.from.4.hydroxybenzoate..eukaryotic.	-0.032	0.007	8.33E-06	6.06E-05	22
PWY.7383.anaerobic.energy.metabolism..invertebrates.cytosol.	-0.043	0.009	8.89E-06	6.29E-05	51
PWY.5872.ubiquinol.10.biosynthesis..eukaryotic.	-0.028	0.006	9.15E-06	6.37E-05	19
PWY0.162.superpathway.of.pyrimidine.ribonucleotides.de.novo.biosynthesis	-0.024	0.005	1.00E-05	6.84E-05	96
SO4ASSIM.PWY.sulfate.reduction.I..assimilatory.	-0.037	0.008	1.03E-05	6.96E-05	81
METSYN.PWY.L.homoserine.and.L.methionine.biosynthesis	-0.018	0.004	1.05E-05	7.00E-05	98
PWY.5347.superpathway.of.L.methionine.biosynthesis..transsulfuration.	-0.018	0.004	1.06E-05	7.03E-05	98
PWY.2723.trehalose.degradation.V	-0.047	0.010	1.07E-05	7.03E-05	82
THISYNARA.PWY.superpathway.of.thiamin.diphosphate.biosynthesis.III..eukaryotes.	-0.023	0.005	1.15E-05	7.43E-05	98
PWY.5189.tetrapyrrole.biosynthesis.II..from.glycine.	-0.024	0.005	1.17E-05	7.45E-05	95
PWY.5870.ubiquinol.8.biosynthesis..eukaryotic.	-0.027	0.006	1.24E-05	7.68E-05	19
PWY.5918.superpathay.of.heme.biosynthesis.from.glutamate	-0.040	0.009	1.26E-05	7.73E-05	88
PANTO.PWY.phosphopantothenate.biosynthesis.I	-0.022	0.005	1.38E-05	8.32E-05	98
PWY.5871.ubiquinol.9.biosynthesis..eukaryotic.	-0.028	0.006	1.42E-05	8.42E-05	19
PWY.5873.ubiquinol.7.biosynthesis..eukaryotic.	-0.028	0.006	1.42E-05	8.42E-05	19
ARGININE.SYN4.PWY.L.ornithine.de.novo.biosynthesis	-0.038	0.008	1.52E-05	8.94E-05	79
PWY.821.superpathway.of.sulfur.amino.acid.biosynthesis..Saccharomyces.cerevisiae	-0.034	0.007	1.58E-05	9.13E-05	70
PANTOSYN.PWY.pantothenate.and.coenzyme.A.biosynthesis.I	-0.021	0.005	1.64E-05	9.37E-05	97
HEME.BIOSYNTHESIS.II.heme.biosynthesis.I..aerobic.	-0.025	0.005	1.93E-05	1.07E-04	97
PWY.6284.superpathway.of.unsaturated.fatty.acids.biosynthesis..E.coli.	-0.047	0.010	2.03E-05	1.11E-04	64
PENTOSE.P.PWY.pentose.phosphate.pathway	-0.020	0.005	2.65E-05	1.36E-04	97
PWY.5188.tetrapyrrole.biosynthesis.I..from.glutamate.	-0.018	0.004	2.99E-05	1.50E-04	95
PWY.5173.superpathway.of.acetyl.CoA.biosynthesis	-0.019	0.004	3.02E-05	1.50E-04	98
PWY.841.superpathway.of.purine.nucleotides.de.novo.biosynthesis.I	-0.017	0.004	3.22E-05	1.58E-04	98
PWY66.399.gluconeogenesis.III	-0.039	0.009	3.51E-05	1.69E-04	47
PWY.7539.6.hydroxymethyl.dihydropterin.diphosphate.biosynthesis.III..Chlamydia.	-0.021	0.005	4.44E-05	2.10E-04	96
PWY.5676.acetyl.CoA.fermentation.to.butanoate.II	-0.038	0.009	4.98E-05	2.33E-04	50
PWY.5941.glycogen.degradation.II..eukaryotic.	-0.024	0.006	5.07E-05	2.35E-04	17
CALVIN.PWY.Calvin.Benson.Bassham.cycle	-0.020	0.005	5.37E-05	2.44E-04	95
PWY.7357.thiamin.formation.from.pyrithiamine.and.oxythiamine..yeast.	-0.021	0.005	5.99E-05	2.69E-04	97
PWY.6147.6.hydroxymethyl.dihydropterin.diphosphate.biosynthesis.I	-0.018	0.004	6.75E-05	2.91E-04	97
SULFATE.CYS.PWY.superpathway.of.sulfate.assimilation.and.cysteine.biosynthesis	-0.037	0.009	6.77E-05	2.91E-04	80
PWY.6897.thiamin.salvage.II	-0.022	0.005	6.86E-05	2.93E-04	95
RIBOSYN2.PWY.flavin.biosynthesis.I..bacteria.and.plants.	-0.021	0.005	7.24E-05	3.04E-04	94
GLYCOLYSIS.glycolysis.I..from.glucose.6.phosphate.	-0.017	0.004	7.59E-05	3.14E-04	98
DAPLYSINESYN.PWY.L.lysine.biosynthesis.I	-0.033	0.008	7.73E-05	3.16E-04	89
PWY.7039.phosphatidate.metabolism.as.a.signaling.molecule	-0.032	0.008	8.13E-05	3.29E-04	25
HISTSYN.PWY.L.histidine.biosynthesis	-0.013	0.003	8.47E-05	3.42E-04	98
PWY.5097.L.lysine.biosynthesis.VI	-0.037	0.009	9.54E-05	3.77E-04	92
PWY.724.superpathway.of.L.lysine.L.threonine.and.L.methionine.biosynthesis.II	-0.036	0.009	1.05E-04	4.11E-04	92
PWY.5367.petroselinate.biosynthesis	-0.040	0.010	1.10E-04	4.19E-04	65
PWY.6859.all.trans.farnesol.biosynthesis	-0.017	0.004	1.13E-04	4.28E-04	97
PWY.5971.palmitate.biosynthesis.II..bacteria.and.plants.	-0.051	0.013	1.25E-04	4.64E-04	64
P4.PWY.superpathway.of.L.lysine.L.threonine.and.L.methionine.biosynthesis.I	-0.032	0.008	1.28E-04	4.72E-04	88
DENITRIFICATION.PWY.nitrate.reduction.I..denitrification.	-0.026	0.007	1.32E-04	4.83E-04	55

Pathway	Coeff	StdErr	p	q	N > 0
TYRFUMCAT.PWY.L.tyrosine.degradation.I	-0.025	0.006	1.33E-04	4.84E-04	59
PWY.7197.pyrimidine.deoxyribonucleotide.phosphorylation	-0.012	0.003	1.35E-04	4.85E-04	98
PWY.7211.superpathway.of.pyrimidine.deoxyribonucleotides.de.novo.biosynthesis	-0.039	0.010	1.42E-04	5.03E-04	74
P124.PWY.Bifidobacterium.shunt	-0.031	0.008	1.54E-04	5.38E-04	92
PWY.5910.superpathway.of.geranylgeranyldiphosphate.biosynthesis.I..via.mevalonate	-0.028	0.007	1.57E-04	5.43E-04	90
HSERMETANA.PWY.L.methionine.biosynthesis.III	-0.020	0.005	1.57E-04	5.43E-04	97
PWY.7208.superpathway.of.pyrimidine.nucleobases.salvage	-0.012	0.003	1.73E-04	5.90E-04	98
GOLPDLCAT.PWY.superpathway.of.glycerol.degradation.to.13.propanediol	-0.036	0.009	1.82E-04	6.16E-04	43
PWY.5080.very.long.chain.fatty.acid.biosynthesis.I	-0.030	0.008	1.86E-04	6.21E-04	22
PWY.6598.sciadonate.biosynthesis	-0.030	0.008	1.86E-04	6.21E-04	22
PWY.7619.juniperonate.biosynthesis	-0.030	0.008	1.86E-04	6.21E-04	22
PWY.4981.L.proline.biosynthesis.II..from.arginine.	-0.017	0.004	1.93E-04	6.40E-04	98
PWY.5484.glycolysis.II..from.fructose.6.phosphate.	-0.019	0.005	2.00E-04	6.62E-04	96
TRPSYN.PWY.L.tryptophan.biosynthesis	-0.016	0.004	2.07E-04	6.78E-04	98
HEXITOLDEGSUPER.PWY.superpathway.of.hexitol.degradation..bacteria.	-0.029	0.007	2.12E-04	6.93E-04	91
PWY.5659.GDP.mannose.biosynthesis	-0.022	0.006	2.18E-04	7.06E-04	95
PWY.6163.chorismate.biosynthesis.from.3.dehydroquinate	-0.011	0.003	2.18E-04	7.06E-04	98
P221.PWY.octane.oxidation	-0.025	0.006	2.21E-04	7.12E-04	87
PWY.6628.superpathway.of.L.phenylalanine.biosynthesis	-0.030	0.008	2.26E-04	7.26E-04	75
ARO.PWY.chorismate.biosynthesis.I	-0.011	0.003	2.33E-04	7.40E-04	98
ARGSYN.PWY.L.arginine.biosynthesis.I..via.L.ornithine.	-0.019	0.005	2.35E-04	7.40E-04	97
COMPLETE.ARO.PWY.superpathway.of.aromatic.amino.acid.biosynthesis	-0.011	0.003	2.38E-04	7.48E-04	98
PWY.7228.superpathway.of.guanosine.nucleotides.de.novo.biosynthesis.I	-0.012	0.003	2.43E-04	7.57E-04	98
ANAEROFRUCAT.PWY.homolactic.fermentation	-0.019	0.005	2.50E-04	7.72E-04	96
PHOSLIPSYN.PWY.superpathway.of.phospholipid.biosynthesis.I..bacteria.	-0.032	0.008	2.64E-04	8.03E-04	90
PWY.2941.L.lysine.biosynthesis.II	-0.021	0.006	2.67E-04	8.06E-04	97
GLCMANNANAUT.PWY.superpathway.of.N.acetylglucosamine.N.acetylmannosamine	-0.026	0.007	2.71E-04	8.10E-04	95
PWY.6168.flavin.biosynthesis.III..fungi.	-0.019	0.005	2.77E-04	8.18E-04	94
ARGSYNBSUB.PWY.L.arginine.biosynthesis.II..acetyl.cycle.	-0.012	0.003	2.80E-04	8.25E-04	98
SER.GLYSYN.PWY.superpathway.of.L.serine.and.glycine.biosynthesis.I	-0.012	0.003	2.88E-04	8.43E-04	98
PWY.6285.superpathway.of.fatty.acids.biosynthesis..E.coli.	-0.042	0.011	2.88E-04	8.43E-04	53
PWY.6125.superpathway.of.guanosine.nucleotides.de.novo.biosynthesis.II	-0.011	0.003	2.96E-04	8.60E-04	98
GLUCOSE1PMETAB.PWY.glucose.and.glucose.1.phosphate.degradation	-0.040	0.011	2.96E-04	8.60E-04	84
PWY0.1298.superpathway.of.pyrimidine.deoxyribonucleosides.degradation	-0.029	0.008	3.02E-04	8.72E-04	91
X1CMET2.PWY.N10.formyl.tetrahydrofolate.biosynthesis	-0.010	0.003	3.07E-04	8.85E-04	98
PWY.2942.L.lysine.biosynthesis.III	-0.011	0.003	3.17E-04	9.06E-04	98
DENOVOPURINE2.PWY.superpathway.of.purine.nucleotides.de.novo.biosynthesis.II	-0.011	0.003	3.22E-04	9.16E-04	98
GLUTORN.PWY.L.ornithine.biosynthesis	-0.021	0.006	3.23E-04	9.16E-04	97
PWY.5855.ubiquinol.7.biosynthesis..prokaryotic.	-0.030	0.008	3.51E-04	9.78E-04	76
PWY.5856.ubiquinol.9.biosynthesis..prokaryotic.	-0.030	0.008	3.51E-04	9.78E-04	76
PWY.5857.ubiquinol.10.biosynthesis..prokaryotic.	-0.030	0.008	3.51E-04	9.78E-04	76
PWY.6708.ubiquinol.8.biosynthesis..prokaryotic.	-0.030	0.008	3.51E-04	9.78E-04	76
FASYN.ELONG.PWY.fatty.acid.elongation.saturated	-0.032	0.009	3.64E-04	1.01E-03	93
POLYISOPRENSYN.PWY.polyisoprenoid.biosynthesis..E.coli.	-0.017	0.004	3.65E-04	1.01E-03	97
GLUCONEO.PWY.gluconeogenesis.I	-0.032	0.009	3.75E-04	1.03E-03	86
PWY.6470.peptidoglycan.biosynthesis.V..beta.lactam.resistance.	-0.020	0.005	3.80E-04	1.04E-03	21
PWY.5514.UDP.N.acetyl.D.galactosamine.biosynthesis.II	-0.021	0.006	3.88E-04	1.06E-03	17
PWY.6588.pyruvate.fermentation.to.acetone	-0.036	0.010	4.14E-04	1.11E-03	54
PWY.6126.superpathway.of.adenosine.nucleotides.de.novo.biosynthesis.II	-0.010	0.003	4.15E-04	1.11E-03	98
COLANSYN.PWY.colanic.acid.building.blocks.biosynthesis	-0.032	0.009	4.19E-04	1.12E-03	61
PWY.7323.superpathway.of.GDP.mannose.derived.O.antigen.building.blocks.biosynth	-0.034	0.009	4.39E-04	1.17E-03	62
PWY.5154.L.arginine.biosynthesis.III..via.N.acetyl.L.citrulline.	-0.031	0.009	4.63E-04	1.20E-03	93
PWY.7282.4.amino.2.methyl.5.phosphomethylpyrimidine.biosynthesis..yeast.	-0.024	0.007	4.77E-04	1.24E-03	91
PWY.7229.superpathway.of.adenosine.nucleotides.de.novo.biosynthesis.I	-0.010	0.003	4.80E-04	1.24E-03	98
NAD.BIOSYNTHESIS.II.NAD.salvage.pathway.II	-0.035	0.010	0.001	0.001	85
PWY.7268.NAD.NADP.NADH.NADPH.cytosolic.interconversion..yeast.	-0.032	0.009	0.001	0.001	65
GLYCOLYSIS.E.D.superpathway.of.glycolysis.and.Entner.Doudoroff	-0.029	0.008	0.001	0.001	88
PWY.5686.UMP.biosynthesis	-0.010	0.003	0.001	0.002	98
PWY.922.mevalonate.pathway.I	-0.028	0.008	0.001	0.002	91
PWY.7288.fatty.acid.beta.oxidation..peroxisome.yeast.	-0.024	0.007	0.001	0.002	56
PWY.7117.C4.photosynthetic.carbon.assimilation.cycle.PEPCK.type	-0.027	0.008	0.001	0.002	94

Pathway	Coeff	StdErr	p	q	N > 0
HISDEG.PWY.L.histidine.degradation.I	-0.016	0.004	0.001	0.002	98
PWY.4984.urea.cycle	-0.029	0.008	0.001	0.002	86
PWY.6385.peptidoglycan.biosynthesis.III..mycobacteria.	-0.011	0.003	0.001	0.002	98
UDPNACETYLGALSYN.PWY.UDP.N.acetyl.D.glucosamine.biosynthesis.II	-0.028	0.008	0.001	0.002	33
PPGPPMET.PWY.ppGpp.biosynthesis	-0.026	0.007	0.001	0.002	74
PWY.6700.queuosine.biosynthesis	-0.011	0.003	0.001	0.002	98
PWY.6124.inosine.5.phosphate.biosynthesis.II	-0.010	0.003	0.001	0.002	98
PWY.3841.folate.transformations.II	-0.010	0.003	0.001	0.002	98
PWY0.781.aspartate.superpathway	-0.028	0.008	0.001	0.002	77
NONOXIPENT.PWY.pentose.phosphate.pathway..non.oxidative.branch.	-0.010	0.003	0.001	0.002	98
POLYAMINSYN3.PWY.superpathway.of.polyamine.biosynthesis.II	-0.029	0.008	0.001	0.002	53
PEPTIDOGLYCANSYN.PWY.peptidoglycan.biosynthesis.I..meso.diaminopimelate.cor	-0.010	0.003	0.001	0.002	98
PWY.5104.L.isoleucine.biosynthesis.IV	-0.020	0.006	0.001	0.002	88
PWY.6471.peptidoglycan.biosynthesis.IV..Enterococcus.faecium.	-0.019	0.006	0.001	0.002	95
PWY.7663.gondoate.biosynthesis..anaerobic.	-0.023	0.007	0.001	0.002	95
UDPNAGSYN.PWY.UDP.N.acetyl.D.glucosamine.biosynthesis.I	-0.010	0.003	0.001	0.002	98
PWY.6282.palmitoleate.biosynthesis.I..from..5Z..dodec.5.enoate.	-0.030	0.009	0.001	0.002	92
OANTIGEN.PWY.O.antigen.building.blocks.biosynthesis..E.coli.	-0.023	0.007	0.001	0.002	95
CITRULBIO.PWY.L.citrulline.biosynthesis	-0.026	0.008	0.001	0.002	87
PWY.7220.adenosine.deoxyribonucleotides.de.novo.biosynthesis.II	-0.010	0.003	0.001	0.002	98
PWY.7222.guanosine.deoxyribonucleotides.de.novo.biosynthesis.II	-0.010	0.003	0.001	0.002	98
PWY.7664.oleate.biosynthesis.IV..anaerobic.	-0.030	0.009	0.001	0.002	92
PWY.5177.glutaryl.CoA.degradation	-0.029	0.009	0.001	0.002	57
PWY.5913.TCA.cycle.VI..obligate.autotrophs.	-0.030	0.009	0.001	0.002	77
PWY66.391.fatty.acid.beta.oxidation.VI..peroxisome.	-0.023	0.007	0.001	0.003	56
PWY.6123.inosine.5.phosphate.biosynthesis.I	-0.009	0.003	0.001	0.003	98
PWY0.862..5Z..dodec.5.enoate.biosynthesis	-0.030	0.009	0.001	0.003	92
PWY.7219.adenosine.ribonucleotides.de.novo.biosynthesis	-0.009	0.003	0.001	0.003	98
PWY.5136.fatty.acid.beta.oxidation.II..peroxisome.	-0.029	0.008	0.001	0.003	87
PWY.7234.inosine.5.phosphate.biosynthesis.III	-0.009	0.003	0.001	0.003	98
GLYCOGENSYNTH.PWY.glycogen.biosynthesis.I..from.ADP.D.Glucose.	-0.016	0.005	0.001	0.003	96
DTDPRHAMSYN.PWY.dTDP.L.rhamnose.biosynthesis.I	-0.020	0.006	0.001	0.003	96
PWY0.1319.CDP.diacylglycerol.biosynthesis.II	-0.009	0.003	0.001	0.003	98
PWY.5667.CDP.diacylglycerol.biosynthesis.I	-0.009	0.003	0.001	0.003	98
PWY.5651.L.tryptophan.degradation.to.2.amino.3.carboxymuconate.semialdehyde	-0.028	0.009	0.001	0.003	26
PWY.7221.guanosine.ribonucleotides.de.novo.biosynthesis	-0.010	0.003	0.001	0.003	98
PWY.4242.pantothenate.and.coenzyme.A.biosynthesis.III	-0.009	0.003	0.001	0.003	98
PWY0.1586.peptidoglycan.maturation..meso.diaminopimelate.containing.	-0.011	0.003	0.001	0.003	98
PWY.6545.pyrimidine.deoxyribonucleotides.de.novo.biosynthesis.III	-0.031	0.009	0.001	0.003	58
PWY.6660.2.heptyl.3.hydroxy.4.1H..quinolone.biosynthesis	-0.022	0.007	0.001	0.003	48
ASPASN.PWY.superpathway.of.L.aspartate.and.L.asparagine.biosynthesis	-0.012	0.004	0.001	0.003	93
PWY.6662.superpathway.of.quinolone.and.alkylquinolone.biosynthesis	-0.022	0.007	0.001	0.003	48
PWY.6612.superpathway.of.tetrahydrofolate.biosynthesis	-0.017	0.005	0.001	0.003	90
PWY.7411.superpathway.of.phosphatidate.biosynthesis..yeast.	-0.013	0.004	0.002	0.003	10
COBALSYN.PWY.adenosylcobalamin.salvage.from.cobinamide.I	-0.033	0.010	0.002	0.003	68
ILEUSYN.PWY.L.isoleucine.biosynthesis.I..from.threonine.	-0.009	0.003	0.002	0.003	98
VALSYN.PWY.L.valine.biosynthesis	-0.009	0.003	0.002	0.003	98
BRANCHED.CHAIN.AA.SYN.PWY.superpathway.of.branched.amino.acid.biosynthesi	-0.009	0.003	0.002	0.003	98
PWY.5695.urate.biosynthesis.inosine.5.phosphate.degradation	-0.010	0.003	0.002	0.003	98
PWY.3001.superpathway.of.L.isoleucine.biosynthesis.I	-0.009	0.003	0.002	0.003	98
PWY.5103.L.isoleucine.biosynthesis.III	-0.009	0.003	0.002	0.003	98
FOLSYN.PWY.superpathway.of.tetrahydrofolate.biosynthesis.and.salvage	-0.016	0.005	0.002	0.004	90
FAO.PWY.fatty.acid.beta.oxidation.I	-0.031	0.010	0.002	0.004	88
PWY.7388.octanoyl..acyl.carrier.protein..biosynthesis..mitochondria.yeast.	-0.024	0.007	0.002	0.004	95
PWY.7400.L.arginine.biosynthesis.IV..archaebacteria.	-0.016	0.005	0.002	0.004	96
THRESYN.PWY.superpathway.of.L.threonine.biosynthesis	-0.009	0.003	0.002	0.004	98
PWY.6737.starch.degradation.V	-0.015	0.005	0.002	0.004	95
COA.PWY.1.coenzyme.A.biosynthesis.II..mammalian.	-0.009	0.003	0.002	0.004	98
PWY.5989.stearate.biosynthesis.II..bacteria.and.plants.	-0.028	0.009	0.002	0.004	92
PWY.7111.pyruvate.fermentation.to.isobutanol..engineered.	-0.009	0.003	0.002	0.004	98
FASYN.INITIAL.PWY.superpathway.of.fatty.acid.biosynthesis.initiation..E.coli.	-0.023	0.007	0.002	0.004	95

Pathway	Coeff	StdErr	p	q	N > 0
COA.PWY.coenzyme.A.biosynthesis.I	-0.009	0.003	0.002	0.004	98
ARGORNPROST.PWY.arginine.ornithine.and.proline.interconversion	-0.020	0.006	0.002	0.005	81
PWY.6629.superpathway.of.L.tryptophan.biosynthesis	-0.022	0.007	0.003	0.005	42
PWY.6122.5.aminoimidazole.ribonucleotide.biosynthesis.II	-0.009	0.003	0.003	0.006	98
PWY.6277.superpathway.of.5.aminoimidazole.ribonucleotide.biosynthesis	-0.009	0.003	0.003	0.006	98
PWY.6703.preQ0.biosynthesis	-0.009	0.003	0.003	0.006	98
PWY.6121.5.aminoimidazole.ribonucleotide.biosynthesis.I	-0.008	0.003	0.003	0.006	98
PWY.6387.UDP.N.acetylmuramoyl.pentapeptide.biosynthesis.I..meso.diaminopimelat	-0.008	0.003	0.003	0.006	98
LEU.DEG2.PWY.L.leucine.degradation.I	-0.025	0.008	0.004	0.007	38
PWY.7118.chitin.degradation.to.ethanol	-0.025	0.008	0.004	0.007	23
PWY.7187.pyrimidine.deoxyribonucleotides.de.novo.biosynthesis.II	-0.015	0.005	0.004	0.007	98
PWY.7184.pyrimidine.deoxyribonucleotides.de.novo.biosynthesis.I	-0.015	0.005	0.004	0.007	98
TRNA.CHARGING.PWY.tRNA.charging	-0.008	0.003	0.004	0.007	98
PWY1G.0.mycothiol.biosynthesis	-0.018	0.006	0.004	0.007	12
PWY.7115.C4.photosynthetic.carbon.assimilation.cycle.NAD.ME.type	-0.026	0.009	0.004	0.007	54
PWY0.1533.methylphosphonate.degradation.I	-0.021	0.007	0.004	0.007	48
PWY.6386.UDP.N.acetylmuramoyl.pentapeptide.biosynthesis.II..lysine.containing.	-0.008	0.003	0.004	0.007	98
NADSYN.PWY.NAD.biosynthesis.II..from.tryptophan.	-0.023	0.008	0.004	0.008	25
PYRIDNUCSYN.PWY.NAD.biosynthesis.I..from.aspartate.	-0.027	0.009	0.004	0.008	76
PWY.6895.superpathway.of.thiamin.diphosphate.biosynthesis.II	-0.031	0.010	0.004	0.008	35
P461.PWY.hexitol.fermentation.to.lactate.formate.ethanol.and.acetate	-0.024	0.008	0.005	0.009	81
PWY.5973.cis.vaccenate.biosynthesis	-0.026	0.009	0.005	0.009	93
GLYOXYLATE.BYPASS.glyoxylate.cycle	-0.035	0.012	0.005	0.010	63
ANAGLYCOLYSIS.PWY.glycolysis.III..from.glucose.	-0.008	0.003	0.006	0.010	98
PWY.6936.seleno.amino.acid.biosynthesis	-0.008	0.003	0.006	0.010	98
TCA.TCA.cycle.I..prokaryotic.	-0.032	0.011	0.006	0.010	71
PWY.4321.L.glutamate.degradation.IV	-0.016	0.006	0.006	0.010	10
PWY1F.823.leucopelargonidin.and.leucocyanidin.biosynthesis	-0.018	0.006	0.006	0.010	25
PWY.7094.fatty.acid.salvage	-0.020	0.007	0.006	0.010	49
PWY.7003.glycerol.degradation.to.butanol	-0.022	0.008	0.006	0.010	34
PWY0.166.superpathway.of.pyrimidine.deoxyribonucleotides.de.novo.biosynthesis..E.	-0.014	0.005	0.006	0.010	98
VALDEG.PWY.L.valine.degradation.I	-0.014	0.005	0.007	0.011	10
TCA.GLYOX.BYPASS.superpathway.of.glyoxylate.bypass.and.TCA	-0.033	0.012	0.007	0.012	63
PWY.7237.myo.chiro.and.scillo.inositol.degradation	-0.030	0.011	0.008	0.013	82
PWY.6318.L.phenylalanine.degradation.IV..mammalian.via.side.chain.	-0.033	0.012	0.009	0.014	77
PWY0.1297.superpathway.of.purine.deoxyribonucleosides.degradation	-0.019	0.007	0.009	0.015	89
PWY.6992.15.anhydrofructose.degradation	-0.013	0.005	0.009	0.015	11
PWY.6562.norspermidine.biosynthesis	-0.006	0.002	0.009	0.015	12
LACTOSECAT.PWY.lactose.and.galactose.degradation.I	-0.009	0.003	0.009	0.015	89
PWY.6891.thiazole.biosynthesis.II..Bacillus.	-0.026	0.010	0.009	0.015	35
PWY66.400.glycolysis.VI..metazoan.	-0.008	0.003	0.009	0.015	98
P125.PWY.superpathway.of..RR..butanediol.biosynthesis	-0.012	0.005	0.010	0.016	92
METHGLYUT.PWY.superpathway.of.methylglyoxal.degradation	-0.031	0.012	0.010	0.016	53
P165.PWY.superpathway.of.purines.degradation.in.plants	-0.021	0.008	0.010	0.016	19
URSIN.PWY.ureide.biosynthesis	-0.018	0.007	0.010	0.016	51
PWY0.1479.tRNA.processing	-0.030	0.012	0.010	0.016	80
PYRIDOXSYN.PWY.pyridoxal.5.phosphate.biosynthesis.I	-0.029	0.011	0.010	0.017	82
PWY0.845.superpathway.of.pyridoxal.5.phosphate.biosynthesis.and.salvage	-0.027	0.011	0.011	0.018	82
GLYCOLYSIS.TCA.GLYOX.BYPASS.superpathway.of.glycolysis.pyruvate.dehydroger	-0.029	0.011	0.012	0.019	63
PWY.7391.isoprene.biosynthesis.II..engineered.	-0.024	0.009	0.012	0.019	61
PWY.7196.superpathway.of.pyrimidine.ribonucleosides.salvage	-0.022	0.009	0.012	0.020	52
PWY.6317.galactose.degradation.I..Leloir.pathway.	-0.017	0.007	0.012	0.020	85
PWY.5265.peptidoglycan.biosynthesis.II..staphylococci.	-0.015	0.006	0.012	0.020	78
PWY66.367.ketogenesis	-0.030	0.012	0.012	0.020	47
PWY.6748.nitrate.reduction.VII..denitrification.	-0.010	0.004	0.012	0.020	36
P441.PWY.superpathway.of.N.acetylneuraminate.degradation	-0.029	0.012	0.013	0.021	75
PWY.241.C4.photosynthetic.carbon.assimilation.cycle.NADP.ME.type	-0.022	0.009	0.015	0.024	55
PWY66.422.D.galactose.degradation.V..Leloir.pathway.	-0.016	0.006	0.015	0.024	91
PWY.6182.superpathway.of.salicylate.degradation	-0.025	0.010	0.015	0.024	65
PWY.6151.S.adenosyl.L.methionine.cycle.I	-0.007	0.003	0.016	0.025	98
PWY.6185.4.methylcatechol.degradation..ortho.cleavage.	-0.024	0.010	0.016	0.025	65

Pathway	Coeff	StdErr	p	q	N > 0
PWY.7269.NAD.NADP.NADH.NADPH.mitochondrial.interconversion..yeast.	-0.026	0.011	0.017	0.027	71
PWY.4041.gamma.glutamyl.cycle	-0.018	0.007	0.018	0.028	84
THISYN.PWY.superpathway.of.thiamin.diphosphate.biosynthesis.I	-0.028	0.012	0.019	0.029	46
PWY.6969.TCA.cycle.V..2.oxoglutarate.ferredoxin.oxidoreductase.	-0.028	0.012	0.020	0.031	51
PWY.6608.guanosine.nucleotides.degradation.III	-0.024	0.010	0.020	0.031	83
PWY.1042.glycolysis.IV..plant.cytosol.	-0.007	0.003	0.021	0.032	98
PWY.6609.adenine.and.adenosine.salvage.III	-0.007	0.003	0.021	0.033	98
NAGLIPASYN.PWY.lipid.IVA.biosynthesis	-0.021	0.009	0.022	0.033	79
PWY.3801.sucrose.degradation.II..sucrose.synthase.	-0.022	0.009	0.022	0.033	64
PWY.6606.guanosine.nucleotides.degradation.II	-0.025	0.011	0.022	0.033	82
PWY3O.355.stearate.biosynthesis.III..fungi.	-0.017	0.008	0.023	0.034	15
PWY.7345.superpathway.of.anaerobic.sucrose.degradation	-0.022	0.009	0.024	0.036	64
PWY.7616.methanol.oxidation.to.carbon.dioxide	-0.019	0.008	0.025	0.037	25
PWY0.1261.anhydromuropeptides.recycling	-0.021	0.009	0.025	0.037	91
PWY.5840.superpathway.of.menaquinol.7.biosynthesis	-0.011	0.005	0.026	0.038	81
PWY.5723.Rubisco.shunt	-0.024	0.011	0.026	0.038	47
PWY.5181.toluene.degradation.III..aerobic...via.p.cresol.	-0.022	0.009	0.026	0.038	61
PRPP.PWY.superpathway.of.histidine.purine.and.pyrimidine.biosynthesis	-0.019	0.009	0.029	0.042	56
UBISYN.PWY.superpathway.of.ubiquinol.8.biosynthesis..prokaryotic.	-0.026	0.011	0.029	0.042	43
PWY.5100.pyruvate.fermentation.to.acetate.and.lactate.II	-0.006	0.003	0.030	0.043	98
CATECHOL.ORTHO.CLEAVAGE.PWY.catechol.degradation.to.beta.ketoadipate	-0.021	0.010	0.030	0.043	67
PWY.5897.superpathway.of.menaquinol.11.biosynthesis	-0.011	0.005	0.030	0.043	81
PWY.5898.superpathway.of.menaquinol.12.biosynthesis	-0.011	0.005	0.030	0.043	81
PWY.5899.superpathway.of.menaquinol.13.biosynthesis	-0.011	0.005	0.030	0.043	81
PWY.5417.catechol.degradation.III..ortho.cleavage.pathway.	-0.022	0.010	0.030	0.043	66
PWY.5431.aromatic.compounds.degradation.via.beta.ketoadipate	-0.022	0.010	0.030	0.043	66
PWY0.1338.polymyxin.resistance	-0.018	0.008	0.030	0.044	58
PWY66.409.superpathway.of.purine.nucleotide.salvage	-0.016	0.007	0.031	0.045	86
PWY.5863.superpathway.of.phylloquinol.biosynthesis	-0.010	0.005	0.031	0.045	81
PWY.7200.superpathway.of.pyrimidine.deoxyribonucleoside.salvage	-0.006	0.003	0.033	0.047	58
P101.PWY.ectoine.biosynthesis	-0.007	0.003	0.034	0.049	17
PWY.7209.superpathway.of.pyrimidine.ribonucleosides.degradation	-0.013	0.006	0.035	0.050	16
PWY0.1241.ADP.L.glycero.beta.D.manno.heptose.biosynthesis	-0.016	0.008	0.037	0.052	57
PROTOCATECHUATE.ORTHO.CLEAVAGE.PWY.protocatechuate.degradation.II..ort	-0.018	0.009	0.038	0.053	65
HEMESYN2.PWY.heme.biosynthesis.II..anaerobic.	-0.018	0.009	0.039	0.055	35
AST.PWY.L.arginine.degradation.II..AST.pathway.	-0.022	0.011	0.040	0.056	66
PWY0.881.superpathway.of.fatty.acid.biosynthesis.I..E.coli.	-0.015	0.007	0.042	0.059	32
PWY.5791.14.dihydroxy.2.naphthoate.biosynthesis.II..plants.	-0.010	0.005	0.044	0.061	81
PWY.5837.14.dihydroxy.2.naphthoate.biosynthesis.I	-0.010	0.005	0.044	0.061	81
PWY.6519.8.amino.7.oxononanoate.biosynthesis.I	-0.020	0.010	0.046	0.063	76
PWY.5838.superpathway.of.menaquinol.8.biosynthesis.I	-0.023	0.012	0.048	0.067	43
KETOGLUCONMET.PWY.ketogluconate.metabolism	-0.018	0.009	0.048	0.067	68
PWY4LZ.257.superpathway.of.fermentation..Chlamydomonas.reinhardtii.	-0.016	0.008	0.049	0.068	53
PWY.6892.thiazole.biosynthesis.I..E.coli.	-0.022	0.011	0.050	0.069	46
BIOTIN.BIOSYNTHESIS.PWY.biotin.biosynthesis.I	-0.020	0.010	0.050	0.069	76
PWY.5861.superpathway.of.demethylmenaquinol.8.biosynthesis	-0.023	0.012	0.055	0.075	43
PWY.6837.fatty.acid.beta.oxidation.V..unsaturated.odd.number.di.isomerase.depende	-0.016	0.008	0.060	0.081	58
PWY.5509.adenosylcobalamin.biosynthesis.from.cobyrinate.ac.diamide.I	-0.006	0.003	0.060	0.082	11
P164.PWY.purine.nucleobases.degradation.I..anaerobic.	-0.011	0.006	0.064	0.086	16
PWY.7245.superpathway.NAD.NADP.NADH.NADPH.interconversion..yeast.	-0.010	0.005	0.071	0.096	14
PWY.7013.L.12.propanediol.degradation	-0.013	0.007	0.072	0.097	29
ARG.POLYAMINE.SYN.superpathway.of.arginine.and.polyamine.biosynthesis	-0.016	0.009	0.072	0.097	61
PWY.7199.pyrimidine.deoxyribonucleosides.salvage	-0.005	0.003	0.074	0.099	98
PWY.1501.mandelate.degradation.I	-0.006	0.003	0.075	0.100	10
PWY.6630.superpathway.of.L.tyrosine.biosynthesis	-0.013	0.007	0.075	0.100	48
PWY.7210.pyrimidine.deoxyribonucleotides.biosynthesis.from.CTP	-0.017	0.009	0.075	0.100	53
PWY4FS.8.phosphatidylglycerol.biosynthesis.II..non.plastidic.	-0.011	0.006	0.075	0.100	95
POLYAMSYN.PWY.superpathway.of.polyamine.biosynthesis.I	-0.017	0.009	0.075	0.101	61
PWY4FS.7.phosphatidylglycerol.biosynthesis.I..plastidic.	-0.011	0.006	0.079	0.104	95
PWY.6305.putrescine.biosynthesis.IV	-0.015	0.009	0.082	0.108	66
LIPASYN.PWY.phospholipases	-0.017	0.010	0.083	0.109	19

Pathway	Coeff	StdErr	p	q	N > 0
GALLATE.DEGRADATION.II.PWY.gallate.degradation.I	-0.007	0.004	0.083	0.109	14
PWY.6595.superpathway.of.guanosine.nucleotides.degradation..plants.	-0.019	0.011	0.087	0.114	81
PWY0.42.2.methylcitrate.cycle.I	-0.013	0.008	0.089	0.117	38
SALVADEHYPOX.PWY.adenosine.nucleotides.degradation.II	-0.015	0.009	0.089	0.117	60
PWY.1269.CMP.3.deoxy.D.manno.octulosonate.biosynthesis.I	-0.017	0.010	0.093	0.121	76
RUMP.PWY.formaldehyde.oxidation.I	-0.005	0.003	0.094	0.122	59
PWY.6113.superpathway.of.mycolate.biosynthesis	-0.004	0.003	0.095	0.124	10
PWY0.1296.purine.ribonucleosides.degradation	-0.005	0.003	0.097	0.125	98
PWY.1861.formaldehyde.assimilation.II..RuMP.Cycle.	-0.009	0.005	0.103	0.133	73
ALLANTOINDEG.PWY.superpathway.of.allantoin.degradation.in.yeast	-0.014	0.008	0.104	0.134	34
P105.PWY.TCA.cycle.IV..2.oxoglutarate.decarboxylase.	-0.015	0.009	0.108	0.140	35
GLUDEG.I.PWY.GABA.shunt	-0.013	0.008	0.111	0.143	23
PWY.6353.purine.nucleotides.degradation.II..aerobic.	-0.014	0.009	0.119	0.152	60
PWY.5675.nitrate.reduction.V..assimilatory.	-0.014	0.009	0.121	0.155	58
PWY.7165.L.ascorbate.biosynthesis.VI..engineered.pathway.	-0.008	0.005	0.127	0.163	17
PWY.7295.L.arabinose.degradation.IV	-0.007	0.004	0.134	0.171	14
PWY.5138.unsaturated.even.numbered.fatty.acid.beta.oxidation	-0.015	0.010	0.138	0.174	71
PWY.5654.2.amino.3.carboxymuconate.semialdehyde.degradation.to.2.oxopentenoat	-0.003	0.002	0.141	0.179	11
PWY.6210.2.aminophenol.degradation	-0.003	0.002	0.141	0.179	11
P42.PWY.incomplete.reductive.TCA.cycle	-0.018	0.013	0.146	0.185	52
PWY.5180.toluene.degradation.I..aerobic...via.o.cresol.	-0.011	0.008	0.151	0.190	24
PWY.5182.toluene.degradation.II..aerobic...via.4.methylcatechol.	-0.011	0.008	0.151	0.190	24
PWYG.321.mycolate.biosynthesis	-0.008	0.006	0.158	0.198	20
PWY.6901.superpathway.of.glucose.and.xylose.degradation	-0.014	0.010	0.165	0.205	42
PWY.4702.phytate.degradation.I	-0.014	0.010	0.168	0.208	65
PWY.6876.isopropanol.biosynthesis	-0.006	0.004	0.171	0.212	15
PWY.5083.NAD.NADH.phosphorylation.and.dephosphorylation	-0.011	0.009	0.183	0.226	61
PWY.561.superpathway.of.glyoxylate.cycle.and.fatty.acid.degradation	-0.014	0.010	0.185	0.228	34
PWY.5005.biotin.biosynthesis.II	-0.005	0.004	0.204	0.250	60
PWY0.1415.superpathway.of.heme.biosynthesis.from.uroporphyrinogen.III	-0.011	0.009	0.205	0.251	29
PWY.6731.starch.degradation.III	-0.012	0.009	0.217	0.264	17
PWY.5415.catechol.degradation.I..meta.cleavage.pathway.	-0.008	0.007	0.226	0.275	25
PWY.7254.TCA.cycle.VII..acetate.producers.	-0.015	0.013	0.243	0.294	39
P122.PWY.heterolactic.fermentation	-0.008	0.007	0.272	0.326	64
PWY3DJ.35471.L.ascorbate.biosynthesis.IV	-0.011	0.010	0.273	0.327	33
PWY.5384.sucrose.degradation.IV..sucrose.phosphorylase.	-0.012	0.011	0.284	0.337	87
PWY.5690.TCA.cycle.II..plants.and.fungi.	-0.014	0.013	0.305	0.361	50
GLUCARDEG.PWY.D.glucarate.degradation.I	-0.011	0.011	0.328	0.385	28
P161.PWY.acetylene.degradation	-0.009	0.010	0.334	0.391	67
PWY.6307.L.tryptophan.degradation.X..mammalian.via.tryptamine.	-0.005	0.006	0.420	0.479	11
PWY0.1061.superpathway.of.L.alanine.biosynthesis	-0.007	0.009	0.453	0.512	64
TEICHOICACID.PWY.teichoic.acid..poly.glycerol..biosynthesis	-0.004	0.005	0.456	0.515	65
ECASYN.PWY.enterobacterial.common.antigen.biosynthesis	0.003	0.004	0.463	0.521	29
RHAMCAT.PWY.L.rhamnose.degradation.I	-0.005	0.007	0.466	0.524	24
PWY.7431.aromatic.biogenic.amine.degradation..bacteria.	-0.005	0.007	0.483	0.541	12
PWY.6313.serotonin.degradation	-0.005	0.007	0.505	0.563	12
PWY.6549.L.glutamine.biosynthesis.III	-0.006	0.009	0.511	0.567	24
REDCITCYC.TCA.cycle.VIII..helicobacter.	-0.007	0.011	0.513	0.569	35
PWY.5028.L.histidine.degradation.II	-0.006	0.009	0.519	0.573	35
P562.PWY.myo.inositol.degradation.I	-0.006	0.009	0.530	0.585	22
GALACTUROCAT.PWY.D.galacturonate.degradation.I	-0.006	0.009	0.532	0.586	16
P621.PWY.nylon.6.oligomer.degradation	-0.003	0.004	0.534	0.587	18
FUCCAT.PWY.fucose.degradation	-0.005	0.008	0.544	0.596	17
P23.PWY.reductive.TCA.cycle.I	-0.005	0.008	0.546	0.597	19
PWY.6507.4.deoxy.L.threo.hex.4.enopyranuronate.degradation	-0.005	0.008	0.548	0.598	47
PWY.621.sucrose.degradation.III..sucrose.invertase.	-0.005	0.008	0.555	0.605	88
KDO.NAGLIPASYN.PWY.superpathway.of..Kdo.2.lipid.A.biosynthesis	-0.003	0.006	0.567	0.616	20
FERMENTATION.PWY.mixed.acid.fermentation	-0.005	0.010	0.586	0.635	51
GALACTARDEG.PWY.D.galactarate.degradation.I	-0.006	0.010	0.594	0.640	19
GLUCARGALACTSUPER.PWY.superpathway.of.D.glucarate.and.D.galactarate.degra	-0.006	0.010	0.594	0.640	19
ENTBACSYN.PWY.enterobactin.biosynthesis	0.004	0.008	0.613	0.657	39

Pathway	Coeff	StdErr	p	q	N > 0
PWY.6531.mannitol.cycle	-0.004	0.009	0.628	0.670	34
PWY.5505.L.glutamate.and.L.glutamine.biosynthesis	0.003	0.007	0.637	0.676	18
PWY.5860.superpathway.of.demethylmenaquinol.6.biosynthesis.I	0.002	0.005	0.676	0.713	30
X3.HYDROXYPHENYLACETATE.DEGRADATION.PWY.4.hydroxyphenylacetate.degı	0.001	0.003	0.701	0.734	16
PWY.6803.phosphatidylcholine.acyl.editing	-0.004	0.011	0.706	0.738	49
PWY.5850.superpathway.of.menaquinol.6.biosynthesis.I	0.002	0.005	0.736	0.764	30
PWY.5896.superpathway.of.menaquinol.10.biosynthesis	0.002	0.005	0.736	0.764	30
PWY.5862.superpathway.of.demethylmenaquinol.9.biosynthesis	0.002	0.005	0.752	0.780	31
PWY.5464.superpathway.of.cytosolic.glycolysis..plants..pyruvate.dehydrogenase.and.	-0.002	0.007	0.783	0.809	11
THREOCAT.PWY.superpathway.of.L.threonine.metabolism	0.001	0.002	0.790	0.814	13
GLUCUROCAT.PWY.superpathway.of.beta.D.glucuronide.and.D.glucuronate.degrada	-0.002	0.007	0.800	0.824	28
PWY0.1277.3.phenylpropanoate.and.3..3.hydroxyphenyl.propanoate.degradation	0.001	0.002	0.810	0.831	13
HCAMHPDEG.PWY.3.phenylpropanoate.and.3..3.hydroxyphenyl.propanoate.degrada	0.001	0.002	0.811	0.831	13
PWY.6690.cinnamate.and.3.hydroxycinnamate.degradation.to.2.oxopent.4.enoate	0.001	0.002	0.811	0.831	13
PWY.5845.superpathway.of.menaquinol.9.biosynthesis	0.001	0.005	0.824	0.844	31
PWY.1541.superpathway.of.taurine.degradation	0.000	0.002	0.848	0.866	13
PWY.7242.D.fructuronate.degradation	0.002	0.009	0.855	0.868	48
ORNDEG.PWY.superpathway.of.ornithine.degradation	0.002	0.009	0.855	0.868	32
ILEUDEG.PWY.L.isoleucine.degradation.I	-0.001	0.006	0.880	0.893	13
PWY.7315.dTDP.N.acetylthomosamine.biosynthesis	0.001	0.010	0.921	0.932	31
PWY5F9.12.biphenyl.degradation	0.000	0.002	0.923	0.933	18
PWY.722.nicotinate.degradation.I	0.001	0.005	0.924	0.933	18
ARGDEG.PWY.superpathway.of.L.arginine.putrescine.and.4.aminobutanoate.degrada	0.000	0.006	0.945	0.949	31
ORNARGDEG.PWY.superpathway.of.L.arginine.and.L.ornithine.degradation	0.000	0.006	0.945	0.949	31
PWY.5022.4.aminobutanoate.degradation.V	-0.001	0.015	0.966	0.968	69
GLYCOL.GLYOXDEG.PWY.superpathway.of.glycol.metabolism.and.degradation	0.000	0.006	0.971	0.972	14
FUC.RHAMCAT.PWY.superpathway.of.fucose.and.rhamnose.degradation	0.000	0.007	0.997	0.997	14
