# Supplementary material for: Supplemental Oxygen Alters the Airway Microbiome in Cystic Fibrosis
Source: mSystems. 2022 Aug 24;7(5):e00364-22. doi: 10.1128/msystems.00364-22 (PMC9601246; doi:10.1128/msystems.00364-22)
Supplement: TABLE S3 [file msystems.00364-22-s0003.pdf]

Pathway	Coeff	StdErr	p	q	N > 0
ANAEROFRUCAT.PWY;homolactic.fermentation	-0.01903	0.004978	0.00025	0.000833	96
ANAGLYCOLYSIS.PWY;glycolysis.III.(from.glucose)	-0.00805	0.00283	0.00559	0.010908	98
DENITRIFICATION.PWY;nitrate.reduction.I.(denitrification)	-0.02648	0.006612	0.000132	0.000503	55
ECASYN.PWY;enterobacterial.common.antigen.biosynthesis	0.002835	0.003844	0.462857	0.544537	29
ENTBACSYN.PWY;enterobactin.biosynthesis	0.004098	0.008062	0.612551	0.655153	39
FERMENTATION.PWY;mixed.acid.fermentation	-0.00546	0.009979	0.585536	0.649024	51
GLYCOCAT.PWY;glycogen.degradation.I.(bacterial)	-0.0442	0.008512	1.40E-06	1.87E-05	84
GLYCOLYSIS.E.D;superpathway.of.glycolysis.and.Entner.Doudoroff	-0.02948	0.008234	0.00057	0.00162	88
GLYOXYLATE.BYPASS;glyoxylate.cycle	-0.03542	0.012401	0.005385	0.010769	63
HEME.BIOSYNTHESIS.II;heme.biosynthesis.I.(aerobic)	-0.02488	0.005494	1.93E-05	0.00011	97
HEMESYN2.PWY;heme.biosynthesis.II.(anaerobic)	-0.0182	0.008681	0.038988	0.05885	35
NAD.BIOSYNTHESIS.II;NAD.salvage.pathway.II	-0.03478	0.009625	0.000511	0.001515	85
NADSYN.PWY;NAD.biosynthesis.II.(from.tryptophan)	-0.02314	0.00787	0.004216	0.009117	25
P105.PWY;TCA.cycle.IV.(2.oxoglutarate.decarboxylase)	-0.0147	0.009059	0.108346	0.14691	35
P122.PWY;heterolactic.fermentation	-0.00827	0.007481	0.272082	0.3455	64
P161.PWY;acetylene.degradation	-0.00928	0.00956	0.334203	0.417754	67
P23.PWY;reductive.TCA.cycle.I	-0.00478	0.007878	0.545785	0.623754	19
P441.PWY;superpathway.of.N.acetylneuraminate.degradation	-0.02934	0.011612	0.013378	0.023267	75
PPGPPMET.PWY;ppGpp.biosynthesis	-0.02616	0.007483	0.000751	0.001826	74
PWY.1861;formaldehyde.assimilation.II.(RuMP.Cycle)	-0.00882	0.005354	0.103062	0.142155	73
PWY.5367;petroselinate.biosynthesis	-0.03985	0.009823	0.00011	0.000439	65
PWY.5392;reductive.TCA.cycle.II	0.002293	0.005141	0.656698	0.691261	10
PWY.5837;14.dihydroxy.2.naphthoate.biosynthesis.I	-0.00957	0.004675	0.043733	0.064789	81
PWY.5897;superpathway.of.menaquinol.11.biosynthesis	-0.01108	0.005011	0.029695	0.04658	81
PWY.6113;superpathway.of.mycolate.biosynthesis	-0.00431	0.002559	0.095495	0.135473	10
PWY.6123;inosine.5.phosphate.biosynthesis.I	-0.00944	0.002802	0.001134	0.002591	98
PWY.6185;4.methylcatechol.degradation.(ortho.cleavage)	-0.0243	0.009912	0.016293	0.027155	65
PWY.6210;2.aminophenol.degradation	-0.00325	0.00219	0.141412	0.188549	11
PWY.6700;queuosine.biosynthesis	-0.0106	0.003031	0.000753	0.001826	98
PWY.6837;fatty.acid.beta.oxidation.V.(unsaturated.odd.number.di.isomerase.depende	-0.01566	0.008204	0.05969	0.086822	58
PWY.7117;C4.photosynthetic.carbon.assimilation.cycle.PEPCK.type	-0.02692	0.007586	0.000635	0.001638	94
PWY.722;nicotinate.degradation.I	0.000509	0.005307	0.92376	0.935453	18
PWY.841;superpathway.of.purine.nucleotides.de.novo.biosynthesis.I	-0.01652	0.003762	3.22E-05	0.000171	98
PWY.922;mevalonate.pathway.I	-0.02799	0.00786	0.000608	0.001622	91
PWY0.1296;purine.ribonucleosides.degradation	-0.00491	0.002919	0.096524	0.135473	98
PWY66.400;glycolysis.VI.(metazoan)	-0.00774	0.002911	0.009387	0.016689	98
PYRIDNUCSAL.PWY;NAD.salvage.pathway.I	-0.03856	0.007304	9.78E-07	1.87E-05	66
PYRIDNUCSYN.PWY;NAD.biosynthesis.I.(from.aspartate)	-0.0274	0.009365	0.004409	0.009281	76
TCA.GLYOX.BYPASS;superpathway.of.glyoxylate.bypass.and.TCA	-0.03276	0.011877	0.007108	0.012923	63
UBISYN.PWY;superpathway.of.ubiquinol.8.biosynthesis.(prokaryotic)	-0.02557	0.011496	0.028777	0.046043	43
