# Supplementary material for: Supplemental Oxygen Alters the Airway Microbiome in Cystic Fibrosis
Source: mSystems. 2022 Aug 24;7(5):e00364-22. doi: 10.1128/msystems.00364-22 (PMC9601246; doi:10.1128/msystems.00364-22)
Supplement: TABLE S4 [file msystems.00364-22-s0004.pdf]

Category	Pathway	Incl	Prev	Criteria
Breakdown.Sugar	ANAGLYCOLYSIS.PWY;glycolysis.III.(from.glucose)	T	98	*Central metabolism sugar breakdown.*
Breakdown.Sugar	GLYCOLYSIS.E.D;superpathway.of.glycolysis.and.Entner.Doudoroff	T	98	*Central metabolism sugar breakdown.*
Fermentation	PWY.5392;reductive.TCA.cycle.II	T	98	*Oxygen-influenced pathway.*
Synthesis.Protein	HEME.BIOSYNTHESIS.II;heme.biosynthesis.I.(aerobic)	T	97	*Oxygen-related pathway.*
Fermentation	ANAEROFRUCAT.PWY;homolactic.fermentation	T	96	*Fermentation reaction.*
Breakdown.Sugar	PWY.5837;14.dihydroxy.2.naphthoate.biosynthesis.I	T	96	*Central metabolism sugar breakdown.*
Synthesis.Lipid	PWY66.400;glycolysis.VI.(metazoan)	T	92	*Oxygen influenced pathway.*
Synthesis.Protein	PWY.6210;2.aminophenol.degradation	T	88	*Oxygen carrier protein.*
Breakdown.Carrier	NAD.BIOSYNTHESIS.II;NAD.salvage.pathway.II	T	85	*Electron transport carrier.*
Breakdown.Sugar	GLYCOCAT.PWY;glycogen.degradation.I.(bacterial)	T	84	*Central metabolism sugar breakdown.*
Fermentation	P441.PWY;superpathway.of.N.acetylneuraminate.degradation	T	81	*Fermentation reaction.*
Synthesis.Carrier	PWY.6113;superpathway.of.mycolate.biosynthesis	T	81	*Electron transport carrier.*
Synthesis.Carrier	PWY.6145;superpathway.of.sialic.acids.and.CMP.sialic.acids.biosyntl	T	81	*Electron transport carrier.*
Respiration	PWY.6185;4.methylcatechol.degradation.(ortho.cleavage)	T	77	*Respiration reaction.*
Synthesis.Carrier	PWY.6123;inosine.5.phosphate.biosynthesis.I	T	76	*Electron transport carrier.*
Synthesis.Carrier	PYRIDNUCSYN.PWY;NAD.biosynthesis.I.(from.aspartate)	T	76	*Electron transport carrier.*
Signaling.Stress	PPGPPMET.PWY;ppGpp.biosynthesis	T	74	*Stress-signaling activity.*
Respiration	TCA.GLYOX.BYPASS;superpathway.of.glyoxylate.bypass.and.TCA	T	71	*Respiration reaction.*
Fermentation	P161.PWY;acetylene.degradation	T	67	*Fermentation reaction.*
Synthesis.Carrier	PYRIDNUCSAL.PWY;NAD.salvage.pathway.I	T	66	*Electron transport carrier.*
Fermentation	P122.PWY;heterolactic.fermentation	T	64	*Fermentation reaction.*
Breakdown.Sugar	PWY0.1296;purine.ribonucleosides.degradation	T	64	*Oxygen influenced pathway.*
Respiration	GLYOXYLATE.BYPASS;glyoxylate.cycle	T	63	*Respiration reaction.*
Synthesis.Antigen	PWY.841;superpathway.of.purine.nucleotides.de.novo.biosynthesis.I	T	62	*Production of antigen compounds.*
Synthesis.Antigen	PWY.922;mevalonate.pathway.I	T	62	*Production of antigen compounds.*
Processing.Carrier	PWY.5367;petroselinate.biosynthesis	T	61	*Electron transport carrier.*
Resistance.Antibiotic	PWY.1861;formaldehyde.assimilation.II.(RuMP.Cycle)	T	58	*Antibiotic resistance process.*
Cycle.Nitrogen	PWY.5897;superpathway.of.menaquinol.11.biosynthesis	T	58	*Oxygen influenced pathway.*
Cycle.Nitrogen	DENITRIFICATION.PWY;nitrate.reduction.I.(denitrification)	T	55	*Oxygen influenced pathway.*
Fermentation	PWY.6837;fatty.acid.beta.oxidation.V.(unsaturated.odd.number.di.isc	T	54	*Fermentation reaction.*
Fermentation	FERMENTATION.PWY;mixed.acid.fermentation	T	51	*Fermentation reaction.*
Respiration	PWY.722;nicotinate.degradation.I	T	51	*Respiration reaction.*
Synthesis.Antibiotic	PWY.7053;docosahexaenoate.biosynthesis.I.(lower.eukaryotes)	T	48	*Antibiotic production process.*
Synthesis.Carrier	UBISYN.PWY;superpathway.of.ubiquinol.8.biosynthesis.(prokaryotic)	T	43	*Electron transport carrier.*
Synthesis.Cofactor	ENTBACSYN.PWY;enterobactin.biosynthesis	T	39	*Oxygen influenced pathway.*
Cycle.Nitrogen	PWY.7117;C4.photosynthetic.carbon.assimilation.cycle.PEPCK.type	T	36	*Oxygen influenced pathway.*
Synthesis.Protein	HEMESYN2.PWY;heme.biosynthesis.II.(anaerobic)	T	35	*Oxygen carrier protein.*
Respiration	P105.PWY;TCA.cycle.IV.(2.oxoglutarate.decarboxylase)	T	35	*Respiration reaction.*
Synthesis.Antigen	ECASYN.PWY;enterobacterial.common.antigen.biosynthesis	T	29	*Production of antigen compounds.*
Synthesis.Carrier	NADSYN.PWY;NAD.biosynthesis.II.(from.tryptophan)	T	25	*Electron transport carrier.*
Signaling.Stress	PWY.7255;ergothioneine.biosynthesis.I.(bacteria)	T	25	*Stress-signaling activity.*
Resistance.Antibiotic	PWY.6700;queuosine.biosynthesis	T	21	*Antibiotic resistance process.*
Respiration	P23.PWY;reductive.TCA.cycle.I	T	19	*Respiration reaction.*
Synthesis.Vitamin	1CMET2.PWY;N10.formyl.tetrahydrofolate.biosynthesis	F	98	*No chemical or reaction of interest.*
Synthesis.Amino	ARGSYN.PWY;L.arginine.biosynthesis.I.(via.L.ornithine)	F	98	*No chemical or reaction of interest.*
Synthesis.Amino	ARO.PWY;chorismate.biosynthesis.I	F	98	*No chemical or reaction of interest.*
Synthesis.Amino	BRANCHED.CHAIN.AA.SYN.PWY;superpathway.of.branched.amino.	F	98	*No chemical or reaction of interest.*
Synthesis.CoA	COA.PWY.1;coenzyme.A.biosynthesis.II.(mammalian)	F	98	*No chemical or reaction of interest.*
Metabolism.Central	COA.PWY;coenzyme.A.biosynthesis.I	F	98	*Human-associated pathway.*
Synthesis.Amino	COMPLETE.ARO.PWY;superpathway.of.aromatic.amino.acid.biosyn	F	98	*No chemical or reaction of interest.*
Synthesis.Nucleic	DENOVOPURINE2.PWY;superpathway.of.purine.nucleotides.de.nov	F	98	*No chemical or reaction of interest.*
Breakdown.Amino	HISDEG.PWY;L.histidine.degradation.I	F	98	*No chemical or reaction of interest.*
Synthesis.Amino	HISTSYN.PWY;L.histidine.biosynthesis	F	98	*No chemical or reaction of interest.*
Synthesis.Amino	HOMOSER.METSYN.PWY;L.methionine.biosynthesis.I	F	98	*No chemical or reaction of interest.*
Synthesis.Amino	ILEUSYN.PWY;L.isoleucine.biosynthesis.I.(from.threonine)	F	98	*No chemical or reaction of interest.*
Synthesis.Amino	METHYLGALLATE.DEGRADATION.PWY;methylgallate.degradation	F	98	*No chemical or reaction of interest.*
Synthesis.Amino	METSYN.PWY;L.homoserine.and.L.methionine.biosynthesis	F	98	*No chemical or reaction of interest.*
Breakdown.Sugar	NONOXIPENT.PWY;pentose.phosphate.pathway.(non.oxidative.bran	F	98	*No chemical or reaction of interest.*
Synthesis.Vitamin	PANTO.PWY;phosphopantothenate.biosynthesis.I	F	98	*No chemical or reaction of interest.*
Synthesis.Structural	PEPTIDOGLYCANSYN.PWY;peptidoglycan.biosynthesis.I.(meso.dia	F	98	*No chemical or reaction of interest.*
Breakdown.Nucleic	PWY.1501;mandelate.degradation.I	F	98	*No chemical or reaction of interest.*
Synthesis.Lipid	PWY.181;photorespiration	F	98	*No chemical or reaction of interest.*
Synthesis.Structural	PWY.241;C4.photosynthetic.carbon.assimilation.cycle.NADP.ME.type	F	98	*No chemical or reaction of interest.*
Synthesis.Nucleic	PWY.2941;L.lysine.biosynthesis.II	F	98	*No chemical or reaction of interest.*
Breakdown.Sugar	PWY.3941;beta.alanine.biosynthesis.II	F	98	*Plant-specific central metabolism sugar breakdown.*
Synthesis.Amino	PWY.5044;purine.nucleotides.degradation.I.(plants)	F	98	*No chemical or reaction of interest.*
Synthesis.Amino	PWY.5055;nicotinate.degradation.III	F	98	*No chemical or reaction of interest.*
Synthesis.Vitamin	PWY.5088;L.glutamate.degradation.VIII.(to.propanoate)	F	98	*No chemical or reaction of interest.*
Synthesis.Cofactor	PWY.5136;fatty.acid.beta.oxidation.II.(peroxisome)	F	98	*No chemical or reaction of interest.*
Synthesis.Amino	PWY.5178;toluene.degradation.IV.(aerobic).(via.catechol)	F	98	*No chemical or reaction of interest.*
Synthesis.Amino	PWY.5417;catechol.degradation.III.(ortho.cleavage.pathway)	F	98	*No chemical or reaction of interest.*
Synthesis.Amino	PWY.5419;catechol.degradation.to.2.oxopent.4.enoate.II	F	98	*No chemical or reaction of interest.*

Category	Pathway	Incl	Prev	Criteria
Synthesis.CoA	PWY.5508;adenosylcobalamin.biosynthesis.from.cobyrinate.ac.diami	F	98	*No chemical or reaction of interest.*
Synthesis.Amino	PWY.5686;UMP.biosynthesis	F	98	*No chemical or reaction of interest.*
Synthesis.Lipid	PWY.5896;superpathway.of.menaquinol.10.biosynthesis	F	98	*No chemical or reaction of interest.*
Synthesis.Nucleic	PWY.5910;superpathway.of.geranylgeranyldiphosphate.biosynthesis.	F	98	*No chemical or reaction of interest.*
Breakdown.Nucleic	PWY.5920;superpathway.of.heme.biosynthesis.from.glycine	F	98	*No chemical or reaction of interest.*
Synthesis.Nucleic	PWY.6309;L.tryptophan.degradation.XI.(mammalian.via.kynurenine)	F	98	*No chemical or reaction of interest.*
Synthesis.Nucleic	PWY.6313;serotonin.degradation	F	98	*No chemical or reaction of interest.*
Synthesis.Nucleic	PWY.6317;galactose.degradation.I.(Leloir.pathway)	F	98	*No chemical or reaction of interest.*
Synthesis.Nucleic	PWY.6318;L.phenylalanine.degradation.IV.(mammalian.via.side.chair	F	98	*No chemical or reaction of interest.*
Synthesis.Nucleic	PWY.6338;superpathway.of.vanillin.and.vanillate.degradation	F	98	*No chemical or reaction of interest.*
Synthesis.Nucleic	PWY.6339;syringate.degradation	F	98	*No chemical or reaction of interest.*
Cycle.SAM	PWY.6383;mono.trans.poly.cis.decaprenyl.phosphate.biosynthesis	F	98	*No chemical or reaction of interest.*
Synthesis.Chemical	PWY.6385;peptidoglycan.biosynthesis.III.(mycobacteria)	F	98	*No chemical or reaction of interest.*
Synthesis.Nucleic	PWY.6507;4.deoxy.L.threo.hex.4.enopyranuronate.degradation	F	98	*No chemical or reaction of interest.*
Synthesis.Structural	PWY.6629;superpathway.of.L.tryptophan.biosynthesis	F	98	*No chemical or reaction of interest.*
Synthesis.Structural	PWY.6630;superpathway.of.L.tyrosine.biosynthesis	F	98	*No chemical or reaction of interest.*
Synthesis.Structural	PWY.6632;caffeine.degradation.IV.(bacteria.via.demethylation.and.o	F	98	*No chemical or reaction of interest.*
Breakdown.Nucleic	PWY.6901;superpathway.of.glucose.and.xylose.degradation	F	98	*No chemical or reaction of interest.*
Breakdown.Sugar	PWY.7013;L.12.propanediol.degradation	F	98	*Human-associated pathway.*
Synthesis.Nucleic	PWY.7090;UDP.23.diacetamido.23.dideoxy.alpha.D.mannuronate.bic	F	98	*No chemical or reaction of interest.*
Synthesis.Nucleic	PWY.7094;fatty.acid.salvage	F	98	*No chemical or reaction of interest.*
Synthesis.Amino	PWY.7219;adenosine.ribonucleotides.de.novo.biosynthesis	F	98	*No chemical or reaction of interest.*
Engineered	PWY.7295;L.arabinose.degradation.IV	F	98	*Artificially engineered pathway.*
Synthesis.Nucleic	PWY.7337;10.cis.heptadecenoyl.CoA.degradation.(yeast)	F	98	*No chemical or reaction of interest.*
Synthesis.Nucleic	PWY.7338;10.trans.heptadecenoyl.CoA.degradation.(reductase.depe	F	98	*No chemical or reaction of interest.*
Breakdown.Nucleic	PWY.7345;superpathway.of.anaerobic.sucrose.degradation	F	98	*No chemical or reaction of interest.*
Breakdown.Nucleic	PWY.7357;thiamin.formation.from.pyrithiamine.and.oxythiamine.(yea	F	98	*No chemical or reaction of interest.*
Breakdown.Nucleic	PWY.7374;14.dihydroxy.6.naphthoate.biosynthesis.I	F	98	*No chemical or reaction of interest.*
Synthesis.Nucleic	PWY.7385;13.propanediol.biosynthesis.(engineered)	F	98	*No chemical or reaction of interest.*
Synthesis.Nucleic	PWY.7389;superpathway.of.anaerobic.energy.metabolism.(invertebra	F	98	*No chemical or reaction of interest.*
Synthesis.Nucleic	PWY.7391;isoprene.biosynthesis.II.(engineered)	F	98	*No chemical or reaction of interest.*
Synthesis.Nucleic	PWY.7392;taxadiene.biosynthesis.(engineered)	F	98	*No chemical or reaction of interest.*
Synthesis.Nucleic	PWY.7397;naringenin.biosynthesis.(engineered)	F	98	*No chemical or reaction of interest.*
Synthesis.Nucleic	PWY.7399;methylphosphonate.degradation.II	F	98	*No chemical or reaction of interest.*
Synthesis.Nucleic	PWY.7400;L.arginine.biosynthesis.IV.(archaebacteria)	F	98	*No chemical or reaction of interest.*
Synthesis.Nucleic	PWY66.422;D.galactose.degradation.V.(Leloir.pathway)	F	98	*No chemical or reaction of interest.*
Synthesis.Amino	SER.GLYSYN.PWY;superpathway.of.L.serine.and.glycine.biosynthes	F	98	*No chemical or reaction of interest.*
Synthesis.Vitamin	THISYN.PWY;superpathway.of.thiamin.diphosphate.biosynthesis.I	F	98	*No chemical or reaction of interest.*
Synthesis.Amino	THRESYN.PWY;superpathway.of.L.threonine.biosynthesis	F	98	*No chemical or reaction of interest.*
Nucleic.Processing	TRNA.CHARGING.PWY;tRNA.charging	F	98	*No chemical or reaction of interest.*
Synthesis.Amino	TRPSYN.PWY;L.tryptophan.biosynthesis	F	98	*No chemical or reaction of interest.*
Synthesis.Nucleic	UDPNAGSYN.PWY;UDP.N.acetyl.D.glucosamine.biosynthesis.I	F	98	*No chemical or reaction of interest.*
Synthesis.Amino	VALSYN.PWY;L.valine.biosynthesis	F	98	*No chemical or reaction of interest.*
Synthesis.Amino	ARGSYNBSUB.PWY;L.arginine.biosynthesis.II.(acetyl.cycle)	F	97	*No chemical or reaction of interest.*
Synthesis.Amino	GLUTORN.PWY;L.ornithine.biosynthesis	F	97	*No chemical or reaction of interest.*
Synthesis.Amino	HSERMETANA.PWY;L.methionine.biosynthesis.III	F	97	*No chemical or reaction of interest.*
Synthesis.Cofactor	PANTOSYN.PWY;pantothenate.and.coenzyme.A.biosynthesis.I	F	97	*No chemical or reaction of interest.*
Breakdown.Sugar	PENTOSE.P.PWY;pentose.phosphate.pathway	F	97	*No chemical or reaction of interest.*
Synthesis.Chemical	POLYISOPRENSYN.PWY;polyisoprenoid.biosynthesis.(E.coli)	F	97	*No chemical or reaction of interest.*
Synthesis.Amino	PWY.5030;L.histidine.degradation.III	F	97	*No chemical or reaction of interest.*
Synthesis.Vitamin	PWY.6352;3.phosphoinositide.biosynthesis	F	97	*No chemical or reaction of interest.*
Synthesis.Chemical	PWY.7199;pyrimidine.deoxyribonucleosides.salvage	F	97	*No chemical or reaction of interest.*
Synthesis.Vitamin	PWY0.1298;superpathway.of.pyrimidine.deoxyribonucleosides.degra	F	97	*No chemical or reaction of interest.*
Synthesis.Nucleic	DTDPRHAMSYN.PWY;dTDP.L.rhamnose.biosynthesis.I	F	96	*No chemical or reaction of interest.*
Synthesis.Carbohydrate	GLYCOGENSYNTH.PWY;glycogen.biosynthesis.I.(from.ADP.D.Gluc	F	96	*No chemical or reaction of interest.*
Synthesis.Nucleic	PWY.2723;trehalose.degradation.V	F	96	*No chemical or reaction of interest.*
Breakdown.Alcohol	PWY.6992;15.anhydrofructose.degradation	F	96	*No chemical or reaction of interest.*
Synthesis.Amino	PWY0.881;superpathway.of.fatty.acid.biosynthesis.I.(E.coli)	F	96	*No chemical or reaction of interest.*
Synthesis.Vitamin	PWY4LZ.257;superpathway.of.fermentation.(Chlamydomonas.reinha	F	96	*No chemical or reaction of interest.*
Cycle.Carbon	CALVIN.PWY;Calvin.Benson.Bassham.cycle	F	95	*No chemical or reaction of interest.*
Synthesis.Lipid	FASYN.INITIAL.PWY;superpathway.of.fatty.acid.biosynthesis.initiatio	F	95	*No chemical or reaction of interest.*
Breakdown.Amino	GLCMANNANAUT.PWY;superpathway.of.N.acetylglucosamine.N.ace	F	95	*No chemical or reaction of interest.*
Synthesis.Antigen	OANTIGEN.PWY;O.antigen.building.blocks.biosynthesis.(E.coli)	F	95	*Narrow-scope production of antigen compounds.*
Synthesis.Lipid	PWY.5180;toluene.degradation.I.(aerobic).(via.o.cresol)	F	95	*No chemical or reaction of interest.*
Synthesis.Lipid	PWY.5181;toluene.degradation.III.(aerobic).(via.p.cresol)	F	95	*No chemical or reaction of interest.*
Synthesis.Cofactor	PWY.5651;L.tryptophan.degradation.to.2.amino.3.carboxymuconate.s	F	95	*No chemical or reaction of interest.*
Synthesis.Cofactor	PWY.5654;2.amino.3.carboxymuconate.semialdehyde.degradation.to	F	95	*No chemical or reaction of interest.*
Synthesis.Nucleic	PWY.5873;ubiquinol.7.biosynthesis.(eukaryotic)	F	95	*No chemical or reaction of interest.*
Synthesis.Structural	PWY.6703;preQ0.biosynthesis	F	95	*No chemical or reaction of interest.*
Breakdown.Carbohydrate	PWY.7115;C4.photosynthetic.carbon.assimilation.cycle.NAD.ME.type	F	95	*No chemical or reaction of interest.*
Breakdown.Vitamin	PWY.7210;pyrimidine.deoxyribonucleotides.biosynthesis.from.CTP	F	95	*No chemical or reaction of interest.*
Synthesis.Lipid	PWY0.321;phenylacetate.degradation.I.(aerobic)	F	95	*No chemical or reaction of interest.*

Category	Pathway	Incl	Prev	Criteria
Synthesis.Lipid	PWY66.399;gluconeogenesis.III	F	95	*Excluded for legibility. Oxygen influenced pathway.*
Synthesis.Carrier	PWY.6396;superpathway.of.23.butanediol.biosynthesis	F	94	*Fungi-specific electron transport carrier.*
Cycle.Carbon	PWY.7315;dTDP.N.acetylthomosamine.biosynthesis	F	94	*Plant-specific oxygen influenced pathway.*
Synthesis.Carrier	RIBOSYN2.PWY;flavin.biosynthesis.I.(bacteria.and.plants)	F	94	*Excluded for legibility. Electron transport carrier.*
Synthesis.Amino	ASPASN.PWY;superpathway.of.L.aspartate.and.L.asparagine.biosyn	F	93	*No chemical or reaction of interest.*
Synthesis.Lipid	FASYN.ELONG.PWY;fatty.acid.elongation.saturated	F	93	*No chemical or reaction of interest.*
Synthesis.Ammino	PWY.5505;L.glutamate.and.L.glutamine.biosynthesis	F	93	*No chemical or reaction of interest.*
Synthesis.Lipid	PWY.6263;superpathway.of.menaquinol.8.biosynthesis.II	F	93	*No chemical or reaction of interest.*
Fermentation	P124.PWY;Bifidobacterium.shunt	F	92	*Narrow-scope Fermentation reaction.*
Fermentation	P125.PWY;superpathway.of.(RR).butanediol.biosynthesis	F	92	*Excluded for legibility. Fermentation reaction.*
Synthesis.Lipid	PWY.3781;aerobic.respiration.I.(cytochrome.c)	F	92	*Excluded for legibility. Oxygen influenced pathway.*
Synthesis.Amino	PWY.5384;sucrose.degradation.IV.(sucrose.phosphorylase)	F	92	*No chemical or reaction of interest.*
Synthesis.Lipid	PWY.6269;adenosylcobalamin.salvage.from.cobinamide.II	F	92	*No chemical or reaction of interest.*
Synthesis.Lipid	PWY.6527;stachyose.degradation	F	92	*No chemical or reaction of interest.*
Synthesis.Amino	PWY.7420;monoacylglycerol.metabolism.(yeast)	F	92	*No chemical or reaction of interest.*
Breakdown.Alcohol	HEXITOLDEGSUPER.PWY;superpathway.of.hexitol.degradation.(bac	F	91	*No chemical or reaction of interest.*
Breakdown.Structural	PWY.1269;CMP.3.deoxy.D.manno.octulosonate.biosynthesis.I	F	91	*No chemical or reaction of interest.*
Breakdown.Nucleic	PWY.1622;formaldehyde.assimilation.I.(serine.pathway)	F	91	*No chemical or reaction of interest.*
Breakdown.Sugar	PWY.7039;phosphatidate.metabolism.as.a.signaling.molecule	F	91	*No chemical or reaction of interest.*
Synthesis.Nucleic	PWY.7560;methylerythritol.phosphate.pathway.II	F	91	*No chemical or reaction of interest.*
Synthesis.Chemical	PWY6666.2;dopamine.degradation	F	91	*No chemical or reaction of interest.*
Synthesis.Vitamin	FOLSYN.PWY;superpathway.of.tetrahydrofolate.biosynthesis.and.sal	F	90	*No chemical or reaction of interest.*
Synthesis.Lipid	PHOSLIPSYN.PWY;superpathway.of.phospholipid.biosynthesis.I.(bac	F	90	*No chemical or reaction of interest.*
Respiration	PWY.5081;L.tryptophan.degradation.VIII.(to.tryptophol)	F	90	*Eukaryote-associated respiration reaction.*
Synthesis.Chemical	PWY.6182;superpathway.of.salicylate.degradation	F	90	*No chemical or reaction of interest.*
Synthesis.Vitamin	PWY.6906;chitin.derivatives.degradation	F	90	*No chemical or reaction of interest.*
Synthesis.Amino	DAPLYSINESYN.PWY;L.lysine.biosynthesis.I	F	89	*No chemical or reaction of interest.*
Breakdown.Sugar	LACTOSECAT.PWY;lactose.and.galactose.degradation.I	F	89	*No chemical or reaction of interest.*
Breakdown.Nucleic	PWY.1541;superpathway.of.taurine.degradation	F	89	*No chemical or reaction of interest.*
Breakdown.Lipid	FAO.PWY;fatty.acid.beta.oxidation.I	F	88	*No chemical or reaction of interest.*
Synthesis.Amino	P461.PWY;hexitol.fermentation.to.lactate.formate.ethanol.and.acetate	F	88	*No chemical or reaction of interest.*
Breakdown.Sugar	PWY.6467;Kdo.transfer.to.lipid.IVA.III.(Chlamydia)	F	88	*No chemical or reaction of interest.*
Synthesis.Amino	CITRULBIO.PWY;L.citrulline.biosynthesis	F	87	*No chemical or reaction of interest.*
Breakdown.Carbohydrate	P221.PWY;octane.oxidation	F	87	*No chemical or reaction of interest.*
Breakdown.Lipid	PWY.5464;superpathway.of.cytosolic.glycolysis.(plants).pyruvate.deh	F	87	*No chemical or reaction of interest.*
Breakdown.Sugar	PWY.5695;urate.biosynthesis.inosine.5.phosphate.degradation	F	87	*No chemical or reaction of interest.*
Synthesis.Protein	PWY.6215;4.chlorobenzoate.degradation	F	87	*Excluded for legibility. Oxygen carrier protein.*
Respiration	PWY.7546;diphthamide.biosynthesis.(eukaryotes)	F	87	*Yeast-specific respiration reaction.*
Synthesis.Sugar	GLUCONEO.PWY;gluconeogenesis.I	F	86	*No chemical or reaction of interest.*
Cycle.Nitrogen	PWY.5179;toluene.degradation.V.(aerobic).(via.toluene.cis.diol)	F	86	*No chemical or reaction of interest.*
Breakdown.Nucleic	PWY.7031;protein.N.glycosylation.(bacterial)	F	86	*No chemical or reaction of interest.*
Breakdown.Sugar	PWY.6588;pyruvate.fermentation.to.acetone	F	85	*No chemical or reaction of interest.*
Breakdown.Sugar	GLUCOSE1PMETAB.PWY;glucose.and.glucose.1.phosphate.degrad	F	84	*No chemical or reaction of interest.*
Cycle.ROS	PWY.5109;2.methylbutanoate.biosynthesis	F	84	*Excluded for legibility. Oxygen influenced pathway.*
Breakdown.Nucleic	PWY.6897;thiamin.salvage.II	F	83	*No chemical or reaction of interest.*
Synthesis.Vitamin	PWY.3661;glycine.betaine.degradation.I	F	82	*No chemical or reaction of interest.*
Breakdown.Sugar	PWY.5028;L.histidine.degradation.II	F	82	*No chemical or reaction of interest.*
Breakdown.Nucleic	PWY.6895;superpathway.of.thiamin.diphosphate.biosynthesis.II	F	82	*No chemical or reaction of interest.*
Breakdown.Alcohol	PWY.7411;superpathway.of.phosphatidate.biosynthesis.(yeast)	F	82	*No chemical or reaction of interest.*
Synthesis.Vitamin	PYRIDOXSYN.PWY;pyridoxal.5.phosphate.biosynthesis.I	F	82	*No chemical or reaction of interest.*
Synthesis.Amino	ARGORNPROST.PWY;arginine.ornithine.and.proline.interconversion	F	81	*No chemical or reaction of interest.*
Synthesis.Carrier	PWY.6075;ergosterol.biosynthesis.I	F	81	*Plant-specific electron transport carrier.*
Synthesis.Carrier	PWY.6098;diploterol.and.cycloartenol.biosynthesis	F	81	*Excluded for legibility. Electron transport carrier.*
Synthesis.Carrier	PWY.6167;flavin.biosynthesis.II.(archaea)	F	81	*Excluded for legibility. Electron transport carrier.*
Synthesis.Carrier	PWY.6168;flavin.biosynthesis.III.(fungi)	F	81	*Excluded for legibility. Electron transport carrier.*
Synthesis.Carrier	PWY.6174;mevalonate.pathway.II.(archaea)	F	81	*Excluded for legibility. Electron transport carrier.*
Breakdown.Nucleic	PWY.6876;isopropanol.biosynthesis	F	81	*No chemical or reaction of interest.*
Synthesis.Chemical	SO4ASSIM.PWY;sulfate.reduction.I.(assimilatory)	F	81	*No chemical or reaction of interest.*
Nucleic.Processing	PWY.2201;folate.transformations.I	F	80	*No chemical or reaction of interest.*
Synthesis.Amino	SULFATE.CYS.PWY;superpathway.of.sulfate.assimilation.and.cystei	F	80	*No chemical or reaction of interest.*
Synthesis.Amino	ARGININE.SYN4.PWY;L.ornithine.de.novo.biosynthesis	F	79	*No chemical or reaction of interest.*
Synthesis.Antigen	NAGLIPASYN.PWY;lipid.IVA.biosynthesis	F	79	*Excluded for legibility. Production of antigen compounds.*
Synthesis.Sugar	PWY.5656;mannosylglycerate.biosynthesis.I	F	78	*Narrow-scope central metabolism energy storage.*
Synthesis.Amino	PWY.5677;succinate.fermentation.to.butanoate	F	78	*No chemical or reaction of interest.*
Synthesis.Amino	PWY.3502;superpathway.of.NAD.biosynthesis.in.eukaryotes	F	77	*No chemical or reaction of interest.*
Breakdown.Amino	PWY.6590;superpathway.of.Clostridium.acetobutylicum.acidogenic.fe	F	77	*No chemical or reaction of interest.*
Breakdown.Sugar	PWY.6760;xylose.degradation.III	F	77	*No chemical or reaction of interest.*
Synthesis.Nucleic	PWY.7347;sucrose.biosynthesis.III	F	77	*No chemical or reaction of interest.*
Synthesis.Cofactor	BIOTIN.BIOSYNTHESIS.PWY;biotin.biosynthesis.I	F	76	*No chemical or reaction of interest.*
Synthesis.Nucleic	PWY.4041;gamma.glutamyl.cycle	F	76	*No chemical or reaction of interest.*
Synthesis.Carrier	PWY.6124;inosine.5.phosphate.biosynthesis.II	F	76	*Excluded for legibility. Electron transport carrier.*
Synthesis.Carrier	PWY.6125;superpathway.of.guanosine.nucleotides.de.novo.biosynthe	F	76	*Excluded for legibility. Electron transport carrier.*

Category	Pathway	Incl	Prev	Criteria
Synthesis.Cofactor	PWY.6749;CMP.legionaminate.biosynthesis.I	F	76	*No chemical or reaction of interest.*
Synthesis.Carrier	PWY.7097;vanillin.and.vanillate.degradation.I	F	76	*Excluded for legibility. Electron transport carrier.*
Breakdown.Chemical	P42.PWY;incomplete.reductive.TCA.cycle	F	75	*No chemical or reaction of interest.*
Synthesis.Amino	PWY.6944;androstenedione.degradation	F	75	*No chemical or reaction of interest.*
Synthesis.Nucleic	PWY.7383;anaerobic.energy.metabolism.(invertebrates.cytosol)	F	74	*No chemical or reaction of interest.*
Cycle.RuMP	PWY.4702;phytate.degradation.I	F	73	*No chemical or reaction of interest.*
Breakdown.Lipid	PWY.5484;glycolysis.II.(from.fructose.6.phosphate)	F	71	*No chemical or reaction of interest.*
Processing.Carrier	PWY.7539;6.hydroxymethyl.dihydropterin.diphosphate.biosynthesis.II	F	71	*Yeast-associated electron transport carrier.*
Synthesis.Amino	PWY66.409;superpathway.of.purine.nucleotide.salvage	F	70	*No chemical or reaction of interest.*
Fermentation	PWY.5189;tetrapyrrole.biosynthesis.II.(from.glycine)	F	69	*Excluded for legibility. Fermentation reaction.*
Synthesis.Cofactor	COBALSYN.PWY;adenosylcobalamin.salvage.from.cobinamide.I	F	68	*No chemical or reaction of interest.*
Breakdown.Sugar	KETOGLUCONMET.PWY;ketogluconate.metabolism	F	68	*No chemical or reaction of interest.*
Breakdown.Chemical	CATECHOL.ORTHO.CLEAVAGE.PWY;catechol.degradation.to.beta.	F	67	*No chemical or reaction of interest.*
Breakdown.Amino	AST.PWY;L.arginine.degradation.II.(AST.pathway)	F	66	*No chemical or reaction of interest.*
Breakdown.Chemical	PWY.5724;superpathway.of.atrazine.degradation	F	66	*No chemical or reaction of interest.*
Breakdown.Chemical	PWY.5754;4.hydroxybenzoate.biosynthesis.I.(eukaryotes)	F	66	*No chemical or reaction of interest.*
Synthesis.Amino	PWY.6549;L.glutamine.biosynthesis.III	F	66	*No chemical or reaction of interest.*
Breakdown.Chemical	PROTOCATECHUATE.ORTHO.CLEAVAGE.PWY;protocatechuate.d	F	65	*No chemical or reaction of interest.*
Breakdown.Chemical	PWY.5156;superpathway.of.fatty.acid.biosynthesis.II.(plant)	F	65	*No chemical or reaction of interest.*
Synthesis.Lipid	PWY.5690;TCA.cycle.II.(plants.and.fungi)	F	65	*No chemical or reaction of interest.*
Breakdown.Chemical	PWY.6433;hydroxylated.fatty.acid.biosynthesis.(plants)	F	65	*No chemical or reaction of interest.*
Breakdown.Chemical	PWY.6435;4.hydroxybenzoate.biosynthesis.V	F	65	*No chemical or reaction of interest.*
Processing.Carrier	PWY.7528;L.methionine.salvage.cycle.I.(bacteria.and.plants)	F	65	*Narrow-scope electron transport carrier.*
Synthesis.Structural	TEICHOICACID.PWY;teichoic.acid.(poly.glycerol).biosynthesis	F	65	*No chemical or reaction of interest.*
Breakdown.Chemical	P185.PWY;formaldehyde.assimilation.III.(dihydroxyacetone.cycle)	F	64	*No chemical or reaction of interest.*
Synthesis.Amino	PWY.101;photosynthesis.light.reactions	F	64	*No chemical or reaction of interest.*
Breakdown.Sugar	PWY.5083;NAD.NADH.phosphorylation.and.dephosphorylation	F	64	*No chemical or reaction of interest.*
Synthesis.Lipid	PWY.622;starch.biosynthesis	F	64	*No chemical or reaction of interest.*
Synthesis.Lipid	PWY.6531;mannitol.cycle	F	64	*No chemical or reaction of interest.*
Respiration	GLYCOLYSIS;glycolysis.I.(from.glucose.6.phosphate)	F	63	*Excluded for legibility. Central metabolism and respiration.*
Bkreakdown.Amino	PWY.5265;peptidoglycan.biosynthesis.II.(staphylococci)	F	63	*No chemical or reaction of interest.*
Respiration	TCA;TCA.cycle.I.(prokaryotic)	F	63	*Excluded for legibility. Respiration reaction.*
Synthesis.Amino	ARG+POLYAMINE.SYN;superpathway.of.arginine.and.polyamine.bio	F	61	*No chemical or reaction of interest.*
Synthesis.Carbohydrate	COLANSYN.PWY;colanic.acid.building.blocks.biosynthesis	F	61	*No chemical or reaction of interest.*
Synthesis.Amino	POLYAMSYN.PWY;superpathway.of.polyamine.biosynthesis.I	F	61	*No chemical or reaction of interest.*
Breakdown.Chemical	PWY.5534;propylene.degradation	F	61	*Excluded for legibility. Oxygen influenced pathway.*
Synthesis.Chemical	PWY0.42;2.methylcitrate.cycle.I	F	61	*Artificially engineered pathway.*
Synthesis.Cofactor	PWY.5188;tetrapyrrole.biosynthesis.I.(from.glutamate)	F	60	*No chemical or reaction of interest.*
Breakdown.Chemical	PWY.5994;palmitate.biosynthesis.I.(animals.and.fungi)	F	60	*No chemical or reaction of interest.*
Breakdown.Nucleic	PWY.6612;superpathway.of.tetrahydrofolate.biosynthesis	F	60	*No chemical or reaction of interest.*
Breakdown.Nucleic	SALVADEHYPOX.PWY;adenosine.nucleotides.degradation.II	F	60	*No chemical or reaction of interest.*
Breakdown.Lipid	PWY.6981;chitin.biosynthesis	F	59	*Human-associated pathway.*
Cycle.RuMP	RUMP.PWY;formaldehyde.oxidation.I	F	59	*No chemical or reaction of interest.*
Breakdown.Amino	TYRFUMCAT.PWY;L.tyrosine.degradation.I	F	59	*No chemical or reaction of interest.*
Synthesis.Nucleic	PWY.6797;6.hydroxymethyl.dihydropterin.diphosphate.biosynthesis.II	F	58	*No chemical or reaction of interest.*
Breakdown.Lipid	PWY.7198;pyrimidine.deoxyribonucleotides.de.novo.biosynthesis.IV	F	58	*No chemical or reaction of interest.*
Breakdown.Nucleic	PWY.7371;14.dihydroxy.6.naphthoate.biosynthesis.II	F	58	*No chemical or reaction of interest.*
Synthesis.Nucleic	PWY.1042;glycolysis.IV.(plant.cytosol)	F	57	*No chemical or reaction of interest.*
Breakdown.Chemical	PWY.5509;adenosylcobalamin.biosynthesis.from.cobyrinate.ac.diami	F	57	*No chemical or reaction of interest.*
Synthesis.Amino	PRPP.PWY;superpathway.of.histidine.purine.and.pyrimidine.biosynth	F	56	*No chemical or reaction of interest.*
Breakdown.Lipid	PWY.6993;nicotine.degradation.II.(pyrrolidine.pathway)	F	56	*Human-associated pathway.*
Breakdown.Lipid	PWY.7606;docosahexaenoate.biosynthesis.III.(mammals)	F	56	*No chemical or reaction of interest.*
Cycle.Carbon	PWY.5022;4.aminobutanoate.degradation.V	F	55	*Plant-specific oxygen influenced pathway.*
Cycle.Carbon	PWY.7312;dTDP.D.beta.fucofuranose.biosynthesis	F	54	*Plant-specific oxygen influenced pathway.*
Breakdown.Chemical	METHANOGENESIS.PWY;methanogenesis.from.H2.and.CO2	F	53	*No chemical or reaction of interest.*
Synthesis.Amino	POLYAMINSYN3.PWY;superpathway.of.polyamine.biosynthesis.II	F	53	*No chemical or reaction of interest.*
Fermentation	PWY.5182;toluene.degradation.II.(aerobic).(via.4.methylcatechol)	F	53	*Narrow-scope Fermentation reaction.*
Synthesis.Lipid	PWY.6545;pyrimidine.deoxyribonucleotides.de.novo.biosynthesis.III	F	53	*No chemical or reaction of interest.*
Synthesis.Nucleic	PWY.7377;cob(II)yrinate.ac.diamide.biosynthesis.I.(early.cobalt.insert	F	53	*No chemical or reaction of interest.*
Respiration	P4.PWY;superpathway.of.L.lysine.L.threonine.and.L.methionine.biosy	F	52	*Excluded for legibility. Respiration reaction.*
Synthesis.Nucleic	PWY.7342;superpathway.of.nicotine.biosynthesis	F	52	*No chemical or reaction of interest.*
Fermentation	PWY0.1586;peptidoglycan.maturation.(meso.diaminopimelate.contair	F	51	*Eukaryote-associated Fermentation reaction.*
Synthesis.Amino	URSIN.PWY;ureide.biosynthesis	F	51	*No chemical or reaction of interest.*
Fermentation	PWY.5898;superpathway.of.menaquinol.12.biosynthesis	F	50	*Excluded for legibility. Fermentation reaction.*
Respiration	PWY.5913;TCA.cycle.VI.(obligate.autotrophs)	F	50	*Plant/Fungi-specific respiration reaction.*
Synthesis.Lipid	PWY.7184;pyrimidine.deoxyribonucleotides.de.novo.biosynthesis.I	F	49	*No chemical or reaction of interest.*
Synthesis.Lipid	PWY.7288;fatty.acid.beta.oxidation.(peroxisome.yeast)	F	49	*No chemical or reaction of interest.*
Breakdown.Chemical	PWY.2221;Entner.Doudoroff.pathway.III.(semi.phosphorylative)	F	48	*No chemical or reaction of interest.*
Synthesis.Amino	PWY.6948;sitosterol.degradation.to.androstenedione	F	48	*No chemical or reaction of interest.*
Synthesis.Antibiotic	PWY.7049;icosapentaenoate.biosynthesis.II.(metazoa)	F	48	*Excluded for legibility. Antibiotic production process.*
Breakdown.Sugar	PWY.7431;aromatic.biogenic.amine.degradation.(bacteria)	F	48	*No chemical or reaction of interest.*
Breakdown.Chemical	PWY.5971;palmitate.biosynthesis.II.(bacteria.and.plants)	F	47	*No chemical or reaction of interest.*

Category	Pathway	Incl	Prev	Criteria
Breakdown.Carbohydrate	PWY.6748;nitrate.reduction.VII.(denitrification)	F	47	*No chemical or reaction of interest.*
Synthesis.Chemical	PWY.6957;mandelate.degradation.to.acetyl.CoA	F	47	*Human-associated pathway.*
Synthesis.Sugar	PWY.7007;methyl.ketone.biosynthesis	F	47	*No chemical or reaction of interest.*
Synthesis.Vitamin	PWY.7208;superpathway.of.pyrimidine.nucleobases.salvage	F	46	*No chemical or reaction of interest.*
Synthesis.Vitamin	THISYNARA.PWY;superpathway.of.thiamin.diphosphate.biosynthesis	F	46	*No chemical or reaction of interest.*
Breakdown.Alcohol	GOLPDLCAT.PWY;superpathway.of.glycerol.degradation.to.13.propa	F	43	*No chemical or reaction of interest.*
Synthesis.Carrier	PWY.6107;chlorosalicylate.degradation	F	43	*Excluded for legibility. Electron transport carrier.*
Synthesis.Carrier	PWY.6138;CMP.N.acetylneuraminate.biosynthesis.I.(eukaryotes)	F	43	*Excluded for legibility. Electron transport carrier.*
Synthesis.Amino	PWY.6945;cholesterol.degradation.to.androstenedione.I.(cholesterol.o	F	42	*No chemical or reaction of interest.*
Breakdown.Sugar	PWY.7211;superpathway.of.pyrimidine.deoxyribonucleotides.de.novo	F	42	*No chemical or reaction of interest.*
Respiration	PWY.7456;mannan.degradation	F	39	*Narrow-scope respiration reaction.*
Breakdown.Amino	LEU.DEG2.PWY;L.leucine.degradation.I	F	38	*No chemical or reaction of interest.*
Breakdown.Carbohydrate	PWY.3481;superpathway.of.L.phenylalanine.and.L.tyrosine.biosynthe	F	38	*No chemical or reaction of interest.*
Breakdown.Amino	PWY.5198;factor.420.biosynthesis	F	35	*No chemical or reaction of interest.*
Synthesis.Vitamin	PWY.7204;pyridoxal.5.phosphate.salvage.II.(plants)	F	35	*No chemical or reaction of interest.*
Synthesis.Vitamin	PWY.7209;superpathway.of.pyrimidine.ribonucleosides.degradation	F	35	*No chemical or reaction of interest.*
Respiration	REDCITCYC;TCA.cycle.VIII.(helicobacter)	F	35	*Narrow-scope respiration reaction.*
Breakdown.Amino	ALL.CHORISMATE.PWY;superpathway.of.chorismate.metabolism	F	34	*No chemical or reaction of interest.*
Synthesis.Carbohydrate	PWY.5861;superpathway.of.demethylmenaquinol.8.biosynthesis	F	34	*No chemical or reaction of interest.*
Breakdown.Alcohol	PWY.6785;hydrogen.production.VIII	F	34	*No chemical or reaction of interest.*
Breakdown.Alcohol	PWY.7238;sucrose.biosynthesis.II	F	34	*No chemical or reaction of interest.*
Synthesis.Vitamin	PWY.5100;pyruvate.fermentation.to.acetate.and.lactate.II	F	33	*No chemical or reaction of interest.*
Synthesis.Nucleic	UDPNACETYLGALSYN.PWY;UDP.N.acetyl.D.glucosamine.biosynthe	F	33	*No chemical or reaction of interest.*
Breakdown.Amino	ORNDEG.PWY;superpathway.of.ornithine.degradation	F	32	*No chemical or reaction of interest.*
Synthesis.Lipid	PWY.3801;sucrose.degradation.II.(sucrose.synthase)	F	32	*No chemical or reaction of interest.*
Synthesis.Amino	ARGDEG.PWY;superpathway.of.L.arginine.putrescine.and.4.aminobu	F	31	*No chemical or reaction of interest.*
Breakdown.Amino	ORNARGDEG.PWY;superpathway.of.L.arginine.and.L.ornithine.degr	F	31	*No chemical or reaction of interest.*
Synthesis.Carrier	PWY.6121;5.aminoimidazole.ribonucleotide.biosynthesis.I	F	31	*Excluded for legibility. Electron transport carrier.*
Synthesis.Carrier	PWY.6143;CMP.pseudaminate.biosynthesis	F	31	*Excluded for legibility. Electron transport carrier.*
Synthesis.Nucleic	PWY.7664;oleate.biosynthesis.IV.(anaerobic)	F	31	*No chemical or reaction of interest.*
Synthesis.Carrier	PWY.6122;5.aminoimidazole.ribonucleotide.biosynthesis.II	F	30	*Excluded for legibility. Electron transport carrier.*
Synthesis.Carrier	PWY.6126;superpathway.of.adenosine.nucleotides.de.novo.biosynthe	F	30	*Excluded for legibility. Electron transport carrier.*
Synthesis.Carrier	PWY.6165;chorismate.biosynthesis.II.(archaea)	F	30	*Excluded for legibility. Electron transport carrier.*
Synthesis.Protein	PWY.1882;superpathway.of.C1.compounds.oxidation.to.CO2	F	29	*Excluded for legibility. Oxygen carrier protein.*
Breakdown.Alcohol	PWY.7245;superpathway.NAD.NADP.NADH.NADPH.interconversion	F	29	*No chemical or reaction of interest.*
Breakdown.Sugar	GLUCARDEG.PWY;D.glucarate.degradation.I	F	28	*No chemical or reaction of interest.*
Breakdown.Sugar	GLUCUROCAT.PWY;superpathway.of.beta.D.glucuronide.and.D.gluc	F	28	*No chemical or reaction of interest.*
Breakdown.Amino	PWY.5863;superpathway.of.phylloquinol.biosynthesis	F	26	*No chemical or reaction of interest.*
Synthesis.Chemical	PWY.4981;L.proline.biosynthesis.II.(from.arginine)	F	25	*No chemical or reaction of interest.*
Breakdown.Chemical	PWY.5723;Rubisco.shunt	F	25	*No chemical or reaction of interest.*
Breakdown.Alcohol	PWY66.389;phytol.degradation	F	25	*No chemical or reaction of interest.*
Breakdown.Chemical	PWY.5532;adenosine.nucleotides.degradation.IV	F	24	*Excluded for legibility. Oxygen influenced pathway.*
Breakdown.Chemical	PWY.561;superpathway.of.glyoxylate.cycle.and.fatty.acid.degradation	F	24	*Excluded for legibility. Oxygen influenced pathway.*
Synthesis.Amino	PWY.6803;phosphatidylcholine.acyl.editing	F	24	*No chemical or reaction of interest.*
Breakdown.Sugar	RHAMCAT.PWY;L.rhamnose.degradation.I	F	24	*No chemical or reaction of interest.*
Breakdown.Chemical	GLUDEG.II.PWY;L.glutamate.degradation.VII.(to.butanoate)	F	23	*No chemical or reaction of interest.*
Breakdown.Structural	PWY.7316;dTDP.N.acetylviosamine.biosynthesis	F	23	*No chemical or reaction of interest.*
Breakdown.Alcohol	P562.PWY;myo.inositol.degradation.I	F	22	*No chemical or reaction of interest.*
Synthesis.Carrier	PWY.5103;L.isoleucine.biosynthesis.III	F	22	*Eukaryote-specific electron transport carrier.*
Synthesis.Lipid	PWY.5345;superpathway.of.L.methionine.biosynthesis.(by.sulfhydryla	F	22	*No chemical or reaction of interest.*
Synthesis.Lipid	PWY.6892;thiazole.biosynthesis.I.(E.coli)	F	22	*No chemical or reaction of interest.*
Synthesis.Lipid	PWY66.391;fatty.acid.beta.oxidation.VI.(peroxisome)	F	22	*No chemical or reaction of interest.*
Synthesis.Lipid	KDO.NAGLIPASYN.PWY;superpathway.of.(Kdo)2.lipid.A.biosynthesis	F	20	*No chemical or reaction of interest.*
Synthesis.Lipid	PWYG.321;mycolate.biosynthesis	F	20	*No chemical or reaction of interest.*
Breakdown.Sugar	GALACT.GLUCUROCAT.PWY;superpathway.of.hexuronide.and.hexu	F	19	*No chemical or reaction of interest.*
Breakdown.Sugar	GLUCARGALACTSUPER.PWY;superpathway.of.D.glucarate.and.D.g	F	19	*No chemical or reaction of interest.*
Breakdown.Lipid	LIPASYN.PWY;phospholipases	F	19	*No chemical or reaction of interest.*
Breakdown.Nucleic	P165.PWY;superpathway.of.purines.degradation.in.plants	F	19	*No chemical or reaction of interest.*
Synthesis.Carrier	PWY.6147;6.hydroxymethyl.dihydropterin.diphosphate.biosynthesis.I	F	19	*Eukaryote-specific electron transport carrier.*
Synthesis.Carrier	PWY.6148;tetrahydromethanopterin.biosynthesis	F	19	*Eukaryote-specific electron transport carrier.*
Synthesis.Carrier	PWY.6151;S.adenosyl.L.methionine.cycle.I	F	19	*Eukaryote-specific electron transport carrier.*
Synthesis.Carrier	PWY.6163;chorismate.biosynthesis.from.3.dehydroquinate	F	19	*Eukaryote-specific electron transport carrier.*
Breakdown.Chemical	P621.PWY;nylon.6.oligomer.degradation	F	18	*No chemical or reaction of interest.*
Synthesis.Amino	PWY.5838;superpathway.of.menaquinol.8.biosynthesis.I	F	18	*No chemical or reaction of interest.*
Breakdown.Chemical	PWY.6277;superpathway.of.5.aminoimidazole.ribonucleotide.biosynth	F	18	*No chemical or reaction of interest.*
Breakdown.Vitamin	PWY.7388;octanoyl.(acyl.carrier.protein).biosynthesis.(mitochondria.)	F	18	*No chemical or reaction of interest.*
Breakdown.Sugar	FUC.RHAMCAT.PWY;superpathway.of.fucose.and.rhamnose.degrad	F	17	*No chemical or reaction of interest.*
Synthesis.Amino	P101.PWY;ectoine.biosynthesis	F	17	*No chemical or reaction of interest.*
Synthesis.Nucleic	PWY.5855;ubiquinol.7.biosynthesis.(prokaryotic)	F	17	*No chemical or reaction of interest.*
Breakdown.Sugar	PWY.621;sucrose.degradation.III.(sucrose.invertase)	F	17	*Eukaryote-specific central metabolism sugar breakdown.*
Breakdown.Carbohydrate	PWY.7111;pyruvate.fermentation.to.isobutanol.(engineered)	F	17	*No chemical or reaction of interest.*
Synthesis.Vitamin	PWY.7332;superpathway.of.UDP.N.acetylglucosamine.derived.O.anti	F	17	*Artificially engineered pathway.*

Category	Pathway	Incl	Prev	Criteria
Breakdown.Chemical	3.HYDROXYPHENYLACETATE.DEGRADATION.PWY;4.hydroxyphe	F	16	*No chemical or reaction of interest.*
Breakdown.Sugar	GALACTUROCAT.PWY;D.galacturonate.degradation.I	F	16	*No chemical or reaction of interest.*
Breakdown.Nucleic	P164.PWY;purine.nucleobases.degradation.I.(anaerobic)	F	16	*No chemical or reaction of interest.*
Breakdown.Nucleic	PWY.7376;cob(II)yrinate.ac.diamide.biosynthesis.II.(late.cobalt.incorp	F	16	*No chemical or reaction of interest.*
Synthesis.Lipid	PWY.5104;L.isoleucine.biosynthesis.IV	F	15	*No chemical or reaction of interest.*
Synthesis.Alcohol	PWY.7200;superpathway.of.pyrimidine.deoxyribonucleoside.salvage	F	15	*Artificially engineered pathway.*
Breakdown.Sugar	FUCCAT.PWY;fucose.degradation	F	14	*No chemical or reaction of interest.*
Breakdown.Chemical	GALLATE.DEGRADATION.I.PWY;gallate.degradation.II	F	14	*No chemical or reaction of interest.*
Breakdown.Chemical	GLYCOL.GLYOXDEG.PWY;superpathway.of.glycol.metabolism.and.c	F	14	*No chemical or reaction of interest.*
Processing.Carrier	PWY.7446;sulfoglycolysis	F	14	*Yeast-specific electron transport carrier.*
Breakdown.Sugar	PWY.7626;bacilysin.biosynthesis	F	14	*No chemical or reaction of interest.*
Breakdown.Alcohol	HCAMHPDEG.PWY;3.phenylpropanoate.and.3.(3.hydroxyphenyl)pro	F	13	*No chemical or reaction of interest.*
Breakdown.Amino	ILEUDEG.PWY;L.isoleucine.degradation.I	F	13	*No chemical or reaction of interest.*
Breakdown.Alcohol	PWY.1361;benzoyl.CoA.degradation.I.(aerobic)	F	13	*No chemical or reaction of interest.*
Breakdown.Amino	PWY.4242;pantothenate.and.coenzyme.A.biosynthesis.III	F	13	*No chemical or reaction of interest.*
Breakdown.Chemical	PWY.7084;nitrifier.denitrification	F	13	*No chemical or reaction of interest.*
Breakdown.Amino	THREOCAT.PWY;superpathway.of.L.threonine.metabolism	F	13	*No chemical or reaction of interest.*
Synthesis.Carrier	PWY.4984;urea.cycle	F	12	*Narrow-scope electron transport carrier.*
Breakdown.Chemical	PWY.6572;chondroitin.sulfate.degradation.I.(bacterial)	F	12	*No chemical or reaction of interest.*
Synthesis.Amino	PWY.6823;molybdenum.cofactor.biosynthesis	F	12	*No chemical or reaction of interest.*
Breakdown.Amino	PWY3O.19;ubiquinol.6.biosynthesis.from.4.hydroxybenzoate.(eukary	F	12	*No chemical or reaction of interest.*
Respiration	PWY.5791;14.dihydroxy.2.naphthoate.biosynthesis.II.(plants)	F	11	*Plant-specific central metabolism and respiration.*
Synthesis.Vitamin	PWY.5850;superpathway.of.menaquinol.6.biosynthesis.I	F	11	*No chemical or reaction of interest.*
Breakdown.Chemical	PWY.5870;ubiquinol.8.biosynthesis.(eukaryotic)	F	11	*No chemical or reaction of interest.*
Breakdown.Alcohol	PWY.6470;peptidoglycan.biosynthesis.V.(beta.lactam.resistance)	F	11	*No chemical or reaction of interest.*
Breakdown.Amino	PWY.6562;norspermidine.biosynthesis	F	11	*No chemical or reaction of interest.*
Breakdown.Sugar	PWY.7235;superpathway.of.ubiquinol.6.biosynthesis.(eukaryotic)	F	11	*No chemical or reaction of interest.*
Breakdown.Chemical	PWY.4221;pantothenate.and.coenzyme.A.biosynthesis.II.(plants)	F	10	*No chemical or reaction of interest.*
Breakdown.Amino	PWY.5138;unsaturated.even.numbered.fatty.acid.beta.oxidation	F	10	*No chemical or reaction of interest.*
Synthesis.Lipid	PWY.6307;L.tryptophan.degradation.X.(mammalian.via.tryptamine)	F	10	*No chemical or reaction of interest.*
Synthesis.Lipid	PWY1G.0;mycothiol.biosynthesis	F	10	*No chemical or reaction of interest.*
Breakdown.Amino	VALDEG.PWY;L.valine.degradation.I	F	10	*No chemical or reaction of interest.*
Prevalence.Excluded	PWY.5514;UDP.N.acetyl.D.galactosamine.biosynthesis.II	F	9	*Prevalence < 10% of cultured samples.*
Prevalence.Excluded	PWY.4202;arsenate.detoxification.I.(glutaredoxin)	F	8	*Prevalence < 10% of cultured samples.*
Prevalence.Excluded	PWY.5845;superpathway.of.menaquinol.9.biosynthesis	F	8	*Prevalence < 10% of cultured samples.*
Prevalence.Excluded	PWY.7409;phospholipid.remodeling.(phosphatidylethanolamine.yeast	F	8	*Prevalence < 10% of cultured samples.*
Prevalence.Excluded	PWY.6641;superpathway.of.sulfolactate.degradation	F	7	*Prevalence < 10% of cultured samples.*
Prevalence.Excluded	PWY.7165;L.ascorbate.biosynthesis.VI.(engineered.pathway)	F	7	*Prevalence < 10% of cultured samples.*
Prevalence.Excluded	CODH.PWY;reductive.acetyl.coenzyme.A.pathway	F	6	*Prevalence < 10% of cultured samples.*
Prevalence.Excluded	GALACTARDEG.PWY;D.galactarate.degradation.I	F	6	*Prevalence < 10% of cultured samples.*
Prevalence.Excluded	PWY.5121;superpathway.of.geranylgeranyl.diphosphate.biosynthesis	F	6	*Prevalence < 10% of cultured samples.*
Prevalence.Excluded	PWY.5862;superpathway.of.demethylmenaquinol.9.biosynthesis	F	6	*Prevalence < 10% of cultured samples.*
Prevalence.Excluded	PWY.6305;putrescine.biosynthesis.IV	F	6	*Prevalence < 10% of cultured samples.*
Prevalence.Excluded	AEROBACTINSYN.PWY;aerobactin.biosynthesis	F	5	*Prevalence < 10% of cultured samples.*
Prevalence.Excluded	PWY.6270;isoprene.biosynthesis.I	F	5	*Prevalence < 10% of cultured samples.*
Prevalence.Excluded	PWY.6608;guanosine.nucleotides.degradation.III	F	5	*Prevalence < 10% of cultured samples.*
Prevalence.Excluded	PWY.7234;inosine.5.phosphate.biosynthesis.III	F	5	*Prevalence < 10% of cultured samples.*
Prevalence.Excluded	PWY0.166;superpathway.of.pyrimidine.deoxyribonucleotides.de.novo	F	5	*Prevalence < 10% of cultured samples.*
Prevalence.Excluded	P108.PWY;pyruvate.fermentation.to.propanoate.I	F	4	*Prevalence < 10% of cultured samples.*
Prevalence.Excluded	PWY.5079;L.phenylalanine.degradation.III	F	4	*Prevalence < 10% of cultured samples.*
Prevalence.Excluded	PWY.5173;superpathway.of.acetyl.CoA.biosynthesis	F	4	*Prevalence < 10% of cultured samples.*
Prevalence.Excluded	PWY.5507;adenosylcobalamin.biosynthesis.I.(early.cobalt.insertion)	F	4	*Prevalence < 10% of cultured samples.*
Prevalence.Excluded	PWY.5705;allantoin.degradation.to.glyoxylate.III	F	4	*Prevalence < 10% of cultured samples.*
Prevalence.Excluded	PWY.7242;D.fructuronate.degradation	F	4	*Prevalence < 10% of cultured samples.*
Prevalence.Excluded	PWY.7268;NAD.NADP.NADH.NADPH.cytosolic.interconversion.(yeas	F	4	*Prevalence < 10% of cultured samples.*
Prevalence.Excluded	PWY.5280;L.lysine.degradation.IV	F	3	*Prevalence < 10% of cultured samples.*
Prevalence.Excluded	PWY.6891;thiazole.biosynthesis.II.(Bacillus)	F	3	*Prevalence < 10% of cultured samples.*
Prevalence.Excluded	PWY.7003;glycerol.degradation.to.butanol	F	3	*Prevalence < 10% of cultured samples.*
Prevalence.Excluded	PWY.7059;fumigaclavine.biosynthesis	F	3	*Prevalence < 10% of cultured samples.*
Prevalence.Excluded	PWY.7282;4.amino.2.methyl.5.phosphomethylpyrimidine.biosynthesis	F	3	*Prevalence < 10% of cultured samples.*
Prevalence.Excluded	ALLANTOINDEG.PWY;superpathway.of.allantoin.degradation.in.yeas	F	2	*Prevalence < 10% of cultured samples.*
Prevalence.Excluded	PWY.5183;superpathway.of.aerobic.toluene.degradation	F	2	*Prevalence < 10% of cultured samples.*
Prevalence.Excluded	PWY.6731;starch.degradation.III	F	2	*Prevalence < 10% of cultured samples.*
Prevalence.Excluded	PWY0.1241;ADP.L.glycero.beta.D.manno.heptose.biosynthesis	F	2	*Prevalence < 10% of cultured samples.*
Prevalence.Excluded	PWY0.1261;anhydromuropeptides.recycling	F	2	*Prevalence < 10% of cultured samples.*
Prevalence.Excluded	PWY.4361;S.methyl.5.thio.alpha.D.ribose.1.phosphate.degradation	F	1	*Prevalence < 10% of cultured samples.*
Prevalence.Excluded	PWY.6598;sciadonate.biosynthesis	F	1	*Prevalence < 10% of cultured samples.*
Prevalence.Excluded	PWY.6728;methylaspartate.cycle	F	1	*Prevalence < 10% of cultured samples.*
Prevalence.Excluded	PWY.6953;dTDP.3.acetamido.36.dideoxy.alpha.D.galactose.biosynth	F	1	*Prevalence < 10% of cultured samples.*
Prevalence.Excluded	PWY.7187;pyrimidine.deoxyribonucleotides.de.novo.biosynthesis.II	F	1	*Prevalence < 10% of cultured samples.*
Prevalence.Excluded	PWY.7286;7.(3.amino.3.carboxypropyl).wyosine.biosynthesis	F	1	*Prevalence < 10% of cultured samples.*
Prevalence.Excluded	PWY.7373;superpathway.of.demethylmenaquinol.6.biosynthesis.II	F	1	*Prevalence < 10% of cultured samples.*

Category	Pathway	Incl	Prev	Criteria
Prevalence.Excluded	PWY0.1061;superpathway.of.L.alanine.biosynthesis	F	1	*Prevalence < 10% of cultured samples.*
Prevalence.Excluded	PWY0.1277;3.phenylpropanoate.and.3.(3.hydroxyphenyl)propanoate.	F	1	*Prevalence < 10% of cultured samples.*
Uncultured.Only	4.HYDROXYMANDELATE.DEGRADATION.PWY;4.hydroxymandelat	F	0	*Observed only in uncultured samples.*
Uncultured.Only	CENTFERM.PWY;pyruvate.fermentation.to.butanoate	F	0	*Observed only in uncultured samples.*
Uncultured.Only	CHLOROPHYLL.SYN;chlorophyllide.a.biosynthesis.I.(aerobic.light.de	F	0	*Observed only in uncultured samples.*
Uncultured.Only	CRNFORCAT.PWY;creatinine.degradation.I	F	0	*Observed only in uncultured samples.*
Uncultured.Only	DHGLUCONATE.PYR.CAT.PWY;glucose.degradation.(oxidative)	F	0	*Observed only in uncultured samples.*
Uncultured.Only	ERGOSTEROL.SYN.PWY;superpathway.of.ergosterol.biosynthesis.I	F	0	*Observed only in uncultured samples.*
Uncultured.Only	GALLATE.DEGRADATION.II.PWY;gallate.degradation.I	F	0	*Observed only in uncultured samples.*
Uncultured.Only	GLUDEG.I.PWY;GABA.shunt	F	0	*Observed only in uncultured samples.*
Uncultured.Only	GLYCOLYSIS.TCA.GLYOX.BYPASS;superpathway.of.glycolysis.pyru	F	0	*Observed only in uncultured samples.*
Uncultured.Only	LPSSYN.PWY;superpathway.of.lipopolysaccharide.biosynthesis	F	0	*Observed only in uncultured samples.*
Uncultured.Only	LYSINE.AMINOAD.PWY;L.lysine.biosynthesis.IV	F	0	*Observed only in uncultured samples.*
Uncultured.Only	LYSINE.DEG1.PWY;L.lysine.degradation.XI.(mammalian)	F	0	*Observed only in uncultured samples.*
Uncultured.Only	MANNOSYL.CHITO.DOLICHOL.BIOSYNTHESIS;protein.N.glycosyla	F	0	*Observed only in uncultured samples.*
Uncultured.Only	MET.SAM.PWY;superpathway.of.S.adenosyl.L.methionine.biosynthes	F	0	*Observed only in uncultured samples.*
Uncultured.Only	METH.ACETATE.PWY;methanogenesis.from.acetate	F	0	*Observed only in uncultured samples.*
Uncultured.Only	METHGLYUT.PWY;superpathway.of.methylglyoxal.degradation	F	0	*Observed only in uncultured samples.*
Uncultured.Only	NONMEVIPP.PWY;methylerythritol.phosphate.pathway.I	F	0	*Observed only in uncultured samples.*
Uncultured.Only	P162.PWY;L.glutamate.degradation.V.(via.hydroxyglutarate)	F	0	*Observed only in uncultured samples.*
Uncultured.Only	P163.PWY;L.lysine.fermentation.to.acetate.and.butanoate	F	0	*Observed only in uncultured samples.*
Uncultured.Only	P184.PWY;protocatechuate.degradation.I.(meta.cleavage.pathway)	F	0	*Observed only in uncultured samples.*
Uncultured.Only	P241.PWY;coenzyme.B.biosynthesis	F	0	*Observed only in uncultured samples.*
Uncultured.Only	P261.PWY;coenzyme.M.biosynthesis.I	F	0	*Observed only in uncultured samples.*
Uncultured.Only	P281.PWY;3.phenylpropanoate.degradation	F	0	*Observed only in uncultured samples.*
Uncultured.Only	PHOTOALL.PWY;oxygenic.photosynthesis	F	0	*Observed only in uncultured samples.*
Uncultured.Only	PROPFERM.PWY;L.alanine.fermentation.to.propanoate.and.acetate	F	0	*Observed only in uncultured samples.*
Uncultured.Only	PWY.2942;L.lysine.biosynthesis.III	F	0	*Observed only in uncultured samples.*
Uncultured.Only	PWY.3001;superpathway.of.L.isoleucine.biosynthesis.I	F	0	*Observed only in uncultured samples.*
Uncultured.Only	PWY.3841;folate.transformations.II	F	0	*Observed only in uncultured samples.*
Uncultured.Only	PWY.4321;L.glutamate.degradation.IV	F	0	*Observed only in uncultured samples.*
Uncultured.Only	PWY.4722;creatinine.degradation.II	F	0	*Observed only in uncultured samples.*
Uncultured.Only	PWY.5004;superpathway.of.L.citrulline.metabolism	F	0	*Observed only in uncultured samples.*
Uncultured.Only	PWY.5005;biotin.biosynthesis.II	F	0	*Observed only in uncultured samples.*
Uncultured.Only	PWY.5067;glycogen.biosynthesis.II.(from.UDP.D.Glucose)	F	0	*Observed only in uncultured samples.*
Uncultured.Only	PWY.5080;very.long.chain.fatty.acid.biosynthesis.I	F	0	*Observed only in uncultured samples.*
Uncultured.Only	PWY.5097;L.lysine.biosynthesis.VI	F	0	*Observed only in uncultured samples.*
Uncultured.Only	PWY.5101;L.isoleucine.biosynthesis.II	F	0	*Observed only in uncultured samples.*
Uncultured.Only	PWY.5129;sphingolipid.biosynthesis.(plants)	F	0	*Observed only in uncultured samples.*
Uncultured.Only	PWY.5154;L.arginine.biosynthesis.III.(via.N.acetyl.L.citrulline)	F	0	*Observed only in uncultured samples.*
Uncultured.Only	PWY.5177;glutaryl.CoA.degradation	F	0	*Observed only in uncultured samples.*
Uncultured.Only	PWY.5304;superpathway.of.sulfur.oxidation.(Acidianus.ambivalens)	F	0	*Observed only in uncultured samples.*
Uncultured.Only	PWY.5306;superpathway.of.thiosulfate.metabolism.(Desulfovibrio.sul	F	0	*Observed only in uncultured samples.*
Uncultured.Only	PWY.5328;superpathway.of.L.methionine.salvage.and.degradation	F	0	*Observed only in uncultured samples.*
Uncultured.Only	PWY.5347;superpathway.of.L.methionine.biosynthesis.(transsulfurati	F	0	*Observed only in uncultured samples.*
Uncultured.Only	PWY.5381;pyridine.nucleotide.cycling.(plants)	F	0	*Observed only in uncultured samples.*
Uncultured.Only	PWY.5415;catechol.degradation.I.(meta.cleavage.pathway)	F	0	*Observed only in uncultured samples.*
Uncultured.Only	PWY.5420;catechol.degradation.II.(meta.cleavage.pathway)	F	0	*Observed only in uncultured samples.*
Uncultured.Only	PWY.5430;meta.cleavage.pathway.of.aromatic.compounds	F	0	*Observed only in uncultured samples.*
Uncultured.Only	PWY.5431;aromatic.compounds.degradation.via.beta.ketoadipate	F	0	*Observed only in uncultured samples.*
Uncultured.Only	PWY.5531;chlorophyllide.a.biosynthesis.II.(anaerobic)	F	0	*Observed only in uncultured samples.*
Uncultured.Only	PWY.5647;2.nitrobenzoate.degradation.I	F	0	*Observed only in uncultured samples.*
Uncultured.Only	PWY.5655;L.tryptophan.degradation.IX	F	0	*Observed only in uncultured samples.*
Uncultured.Only	PWY.5659;GDP.mannose.biosynthesis	F	0	*Observed only in uncultured samples.*
Uncultured.Only	PWY.5667;CDP.diacylglycerol.biosynthesis.I	F	0	*Observed only in uncultured samples.*
Uncultured.Only	PWY.5675;nitrate.reduction.V.(assimilatory)	F	0	*Observed only in uncultured samples.*
Uncultured.Only	PWY.5676;acetyl.CoA.fermentation.to.butanoate.II	F	0	*Observed only in uncultured samples.*
Uncultured.Only	PWY.5692;allantoin.degradation.to.glyoxylate.II	F	0	*Observed only in uncultured samples.*
Uncultured.Only	PWY.5741;ethylmalonyl.CoA.pathway	F	0	*Observed only in uncultured samples.*
Uncultured.Only	PWY.5747;2.methylcitrate.cycle.II	F	0	*Observed only in uncultured samples.*
Uncultured.Only	PWY.5751;phenylethanol.biosynthesis	F	0	*Observed only in uncultured samples.*
Uncultured.Only	PWY.5840;superpathway.of.menaquinol.7.biosynthesis	F	0	*Observed only in uncultured samples.*
Uncultured.Only	PWY.5856;ubiquinol.9.biosynthesis.(prokaryotic)	F	0	*Observed only in uncultured samples.*
Uncultured.Only	PWY.5857;ubiquinol.10.biosynthesis.(prokaryotic)	F	0	*Observed only in uncultured samples.*
Uncultured.Only	PWY.5860;superpathway.of.demethylmenaquinol.6.biosynthesis.I	F	0	*Observed only in uncultured samples.*
Uncultured.Only	PWY.5871;ubiquinol.9.biosynthesis.(eukaryotic)	F	0	*Observed only in uncultured samples.*
Uncultured.Only	PWY.5872;ubiquinol.10.biosynthesis.(eukaryotic)	F	0	*Observed only in uncultured samples.*
Uncultured.Only	PWY.5899;superpathway.of.menaquinol.13.biosynthesis	F	0	*Observed only in uncultured samples.*
Uncultured.Only	PWY.5918;superpathay.of.heme.biosynthesis.from.glutamate	F	0	*Observed only in uncultured samples.*
Uncultured.Only	PWY.5941;glycogen.degradation.II.(eukaryotic)	F	0	*Observed only in uncultured samples.*
Uncultured.Only	PWY.5973;cis.vaccenate.biosynthesis	F	0	*Observed only in uncultured samples.*
Uncultured.Only	PWY.5989;stearate.biosynthesis.II.(bacteria.and.plants)	F	0	*Observed only in uncultured samples.*

Category	Pathway	Incl	Prev	Criteria
Uncultured.Only	PWY.6071;superpathway.of.phenylethylamine.degradation	F	0	*Observed only in uncultured samples.*
Uncultured.Only	PWY.6074;zymosterol.biosynthesis	F	0	*Observed only in uncultured samples.*
Uncultured.Only	PWY.6281;L.selenocysteine.biosynthesis.II.(archaea.and.eukaryotes)	F	0	*Observed only in uncultured samples.*
Uncultured.Only	PWY.6282;palmitoleate.biosynthesis.I.(from.(5Z).dodec.5.enoate)	F	0	*Observed only in uncultured samples.*
Uncultured.Only	PWY.6284;superpathway.of.unsaturated.fatty.acids.biosynthesis.(E.c	F	0	*Observed only in uncultured samples.*
Uncultured.Only	PWY.6285;superpathway.of.fatty.acids.biosynthesis.(E.coli)	F	0	*Observed only in uncultured samples.*
Uncultured.Only	PWY.6342;noradrenaline.and.adrenaline.degradation	F	0	*Observed only in uncultured samples.*
Uncultured.Only	PWY.6349;CDP.archaeol.biosynthesis	F	0	*Observed only in uncultured samples.*
Uncultured.Only	PWY.6351;D.myo.inositol.(145).trisphosphate.biosynthesis	F	0	*Observed only in uncultured samples.*
Uncultured.Only	PWY.6353;purine.nucleotides.degradation.II.(aerobic)	F	0	*Observed only in uncultured samples.*
Uncultured.Only	PWY.6386;UDP.N.acetylmuramoyl.pentapeptide.biosynthesis.II.(lysin	F	0	*Observed only in uncultured samples.*
Uncultured.Only	PWY.6387;UDP.N.acetylmuramoyl.pentapeptide.biosynthesis.I.(meso	F	0	*Observed only in uncultured samples.*
Uncultured.Only	PWY.6415;L.ascorbate.biosynthesis.V	F	0	*Observed only in uncultured samples.*
Uncultured.Only	PWY.6471;peptidoglycan.biosynthesis.IV.(Enterococcus.faecium)	F	0	*Observed only in uncultured samples.*
Uncultured.Only	PWY.6478;GDP.D.glycero.alpha.D.manno.heptose.biosynthesis	F	0	*Observed only in uncultured samples.*
Uncultured.Only	PWY.6486;D.galacturonate.degradation.II	F	0	*Observed only in uncultured samples.*
Uncultured.Only	PWY.6503;superpathway.of.ergotamine.biosynthesis	F	0	*Observed only in uncultured samples.*
Uncultured.Only	PWY.6505;L.tryptophan.degradation.XII.(Geobacillus)	F	0	*Observed only in uncultured samples.*
Uncultured.Only	PWY.6519;8.amino.7.oxononanoate.biosynthesis.I	F	0	*Observed only in uncultured samples.*
Uncultured.Only	PWY.6565;superpathway.of.polyamine.biosynthesis.III	F	0	*Observed only in uncultured samples.*
Uncultured.Only	PWY.6595;superpathway.of.guanosine.nucleotides.degradation.(plan	F	0	*Observed only in uncultured samples.*
Uncultured.Only	PWY.6596;adenosine.nucleotides.degradation.I	F	0	*Observed only in uncultured samples.*
Uncultured.Only	PWY.6606;guanosine.nucleotides.degradation.II	F	0	*Observed only in uncultured samples.*
Uncultured.Only	PWY.6609;adenine.and.adenosine.salvage.III	F	0	*Observed only in uncultured samples.*
Uncultured.Only	PWY.6628;superpathway.of.L.phenylalanine.biosynthesis	F	0	*Observed only in uncultured samples.*
Uncultured.Only	PWY.6654;phosphopantothenate.biosynthesis.III	F	0	*Observed only in uncultured samples.*
Uncultured.Only	PWY.6660;2.heptyl.3.hydroxy.4(1H).quinolone.biosynthesis	F	0	*Observed only in uncultured samples.*
Uncultured.Only	PWY.6662;superpathway.of.quinolone.and.alkylquinolone.biosynthesi	F	0	*Observed only in uncultured samples.*
Uncultured.Only	PWY.6690;cinnamate.and.3.hydroxycinnamate.degradation.to.2.oxop	F	0	*Observed only in uncultured samples.*
Uncultured.Only	PWY.6708;ubiquinol.8.biosynthesis.(prokaryotic)	F	0	*Observed only in uncultured samples.*
Uncultured.Only	PWY.6737;starch.degradation.V	F	0	*Observed only in uncultured samples.*
Uncultured.Only	PWY.6829;tRNA.methylation.(yeast)	F	0	*Observed only in uncultured samples.*
Uncultured.Only	PWY.6834;spermidine.biosynthesis.III	F	0	*Observed only in uncultured samples.*
Uncultured.Only	PWY.6859;all.trans.farnesol.biosynthesis	F	0	*Observed only in uncultured samples.*
Uncultured.Only	PWY.6936;seleno.amino.acid.biosynthesis	F	0	*Observed only in uncultured samples.*
Uncultured.Only	PWY.6969;TCA.cycle.V.(2.oxoglutarate;ferredoxin.oxidoreductase)	F	0	*Observed only in uncultured samples.*
Uncultured.Only	PWY.7036;very.long.chain.fatty.acid.biosynthesis.II	F	0	*Observed only in uncultured samples.*
Uncultured.Only	PWY.7046;4.coumarate.degradation.(anaerobic)	F	0	*Observed only in uncultured samples.*
Uncultured.Only	PWY.7098;vanillin.and.vanillate.degradation.II	F	0	*Observed only in uncultured samples.*
Uncultured.Only	PWY.7118;chitin.degradation.to.ethanol	F	0	*Observed only in uncultured samples.*
Uncultured.Only	PWY.7124;ethylene.biosynthesis.V.(engineered)	F	0	*Observed only in uncultured samples.*
Uncultured.Only	PWY.7159;chlorophyllide.a.biosynthesis.III.(aerobic.light.independent	F	0	*Observed only in uncultured samples.*
Uncultured.Only	PWY.7196;superpathway.of.pyrimidine.ribonucleosides.salvage	F	0	*Observed only in uncultured samples.*
Uncultured.Only	PWY.7197;pyrimidine.deoxyribonucleotide.phosphorylation	F	0	*Observed only in uncultured samples.*
Uncultured.Only	PWY.7218;photosynthetic.3.hydroxybutanoate.biosynthesis.(enginee	F	0	*Observed only in uncultured samples.*
Uncultured.Only	PWY.7220;adenosine.deoxyribonucleotides.de.novo.biosynthesis.II	F	0	*Observed only in uncultured samples.*
Uncultured.Only	PWY.7221;guanosine.ribonucleotides.de.novo.biosynthesis	F	0	*Observed only in uncultured samples.*
Uncultured.Only	PWY.7222;guanosine.deoxyribonucleotides.de.novo.biosynthesis.II	F	0	*Observed only in uncultured samples.*
Uncultured.Only	PWY.7228;superpathway.of.guanosine.nucleotides.de.novo.biosynthe	F	0	*Observed only in uncultured samples.*
Uncultured.Only	PWY.7229;superpathway.of.adenosine.nucleotides.de.novo.biosynthe	F	0	*Observed only in uncultured samples.*
Uncultured.Only	PWY.7237;myo.chiro.and.scillo.inositol.degradation	F	0	*Observed only in uncultured samples.*
Uncultured.Only	PWY.724;superpathway.of.L.lysine.L.threonine.and.L.methionine.bios	F	0	*Observed only in uncultured samples.*
Uncultured.Only	PWY.7254;TCA.cycle.VII.(acetate.producers)	F	0	*Observed only in uncultured samples.*
Uncultured.Only	PWY.7269;NAD.NADP.NADH.NADPH.mitochondrial.interconversion.	F	0	*Observed only in uncultured samples.*
Uncultured.Only	PWY.7279;aerobic.respiration.II.(cytochrome.c).(yeast)	F	0	*Observed only in uncultured samples.*
Uncultured.Only	PWY.7283;wybutosine.biosynthesis	F	0	*Observed only in uncultured samples.*
Uncultured.Only	PWY.7290;Escherichia.coli.serotype.O86.O.antigen.biosynthesis	F	0	*Observed only in uncultured samples.*
Uncultured.Only	PWY.7294;xylose.degradation.IV	F	0	*Observed only in uncultured samples.*
Uncultured.Only	PWY.7323;superpathway.of.GDP.mannose.derived.O.antigen.buildin	F	0	*Observed only in uncultured samples.*
Uncultured.Only	PWY.7328;superpathway.of.UDP.glucose.derived.O.antigen.building.	F	0	*Observed only in uncultured samples.*
Uncultured.Only	PWY.7384;anaerobic.energy.metabolism.(invertebrates.mitochondria	F	0	*Observed only in uncultured samples.*
Uncultured.Only	PWY.7413;dTDP.6.deoxy.alpha.D.allose.biosynthesis	F	0	*Observed only in uncultured samples.*
Uncultured.Only	PWY.7527;L.methionine.salvage.cycle.III	F	0	*Observed only in uncultured samples.*
Uncultured.Only	PWY.7579;phycourobilin.biosynthesis	F	0	*Observed only in uncultured samples.*
Uncultured.Only	PWY.7592;arachidonate.biosynthesis.III.(metazoa)	F	0	*Observed only in uncultured samples.*
Uncultured.Only	PWY.7616;methanol.oxidation.to.carbon.dioxide	F	0	*Observed only in uncultured samples.*
Uncultured.Only	PWY.7619;juniperonate.biosynthesis	F	0	*Observed only in uncultured samples.*
Uncultured.Only	PWY.7663;gondoate.biosynthesis.(anaerobic)	F	0	*Observed only in uncultured samples.*
Uncultured.Only	PWY.821;superpathway.of.sulfur.amino.acid.biosynthesis.(Saccharon	F	0	*Observed only in uncultured samples.*
Uncultured.Only	PWY0.1297;superpathway.of.purine.deoxyribonucleosides.degradatic	F	0	*Observed only in uncultured samples.*
Uncultured.Only	PWY0.1319;CDP.diacylglycerol.biosynthesis.II	F	0	*Observed only in uncultured samples.*
Uncultured.Only	PWY0.1338;polymyxin.resistance	F	0	*Observed only in uncultured samples.*

Category	Pathway	Incl	Prev	Criteria
Uncultured.Only	PWY0.1415;superpathway.of.heme.biosynthesis.from.uroporphyrinog	F	0	*Observed only in uncultured samples.*
Uncultured.Only	PWY0.1479;tRNA.processing	F	0	*Observed only in uncultured samples.*
Uncultured.Only	PWY0.1533;methylphosphonate.degradation.I	F	0	*Observed only in uncultured samples.*
Uncultured.Only	PWY0.162;superpathway.of.pyrimidine.ribonucleotides.de.novo.biosy	F	0	*Observed only in uncultured samples.*
Uncultured.Only	PWY0.41;allantoin.degradation.IV.(anaerobic)	F	0	*Observed only in uncultured samples.*
Uncultured.Only	PWY0.781;aspartate.superpathway	F	0	*Observed only in uncultured samples.*
Uncultured.Only	PWY0.845;superpathway.of.pyridoxal.5.phosphate.biosynthesis.and.s	F	0	*Observed only in uncultured samples.*
Uncultured.Only	PWY0.862;(5Z).dodec.5.enoate.biosynthesis	F	0	*Observed only in uncultured samples.*
Uncultured.Only	PWY1F.823;leucopelargonidin.and.leucocyanidin.biosynthesis	F	0	*Observed only in uncultured samples.*
Uncultured.Only	PWY3DJ.35471;L.ascorbate.biosynthesis.IV	F	0	*Observed only in uncultured samples.*
Uncultured.Only	PWY3O.1109;superpathway.of.4.hydroxybenzoate.biosynthesis.(yeas	F	0	*Observed only in uncultured samples.*
Uncultured.Only	PWY3O.355;stearate.biosynthesis.III.(fungi)	F	0	*Observed only in uncultured samples.*
Uncultured.Only	PWY490.3;nitrate.reduction.VI.(assimilatory)	F	0	*Observed only in uncultured samples.*
Uncultured.Only	PWY4FS.7;phosphatidylglycerol.biosynthesis.I.(plastidic)	F	0	*Observed only in uncultured samples.*
Uncultured.Only	PWY4FS.8;phosphatidylglycerol.biosynthesis.II.(non.plastidic)	F	0	*Observed only in uncultured samples.*
Uncultured.Only	PWY5F9.12;biphenyl.degradation	F	0	*Observed only in uncultured samples.*
Uncultured.Only	PWY66.201;nicotine.degradation.IV	F	0	*Observed only in uncultured samples.*
Uncultured.Only	PWY66.367;ketogenesis	F	0	*Observed only in uncultured samples.*
Uncultured.Only	PWY66.374;C20.prostanoid.biosynthesis	F	0	*Observed only in uncultured samples.*
Uncultured.Only	PWY66.388;fatty.acid.alpha.oxidation.III	F	0	*Observed only in uncultured samples.*
Uncultured.Only	PWY66.398;TCA.cycle.III.(animals)	F	0	*Observed only in uncultured samples.*
Uncultured.Only	SPHINGOLIPID.SYN.PWY;sphingolipid.biosynthesis.(yeast)	F	0	*Observed only in uncultured samples.*
Uncultured.Only	SUCSYN.PWY;sucrose.biosynthesis.I.(from.photosynthesis)	F	0	*Observed only in uncultured samples.*
Uncultured.Only	TRIGLSYN.PWY;diacylglycerol.and.triacylglycerol.biosynthesis	F	0	*Observed only in uncultured samples.*
Uncultured.Only	TRYPTOPHAN.DEGRADATION.1;L.tryptophan.degradation.III.(eukar	F	0	*Observed only in uncultured samples.*
Uncultured.Only	URDEGR.PWY;superpathway.of.allantoin.degradation.in.plants	F	0	*Observed only in uncultured samples.*
