# Supplementary material for: Supplemental Oxygen Alters the Airway Microbiome in Cystic Fibrosis
Source: mSystems. 2022 Aug 24;7(5):e00364-22. doi: 10.1128/msystems.00364-22 (PMC9601246; doi:10.1128/msystems.00364-22)
Supplement: TABLE S5 [file msystems.00364-22-s0005.pdf]

Metric	Normoxia	Hyperoxia	Value	pVal		Description
Adjusted Rand Index			0.462	0.000	***	Chance-adjusted similarity of node pair placements.
Components	1	16	+15	0.001	***	Number of disconnected components in the network.
Cluster Coefficient	0.688	0.841	+0.154	0.002	**	Degree to which nodes cluster together.
Degree Centrality Dissimilarity			0.920	0.004	**	Degree of shift to the average number of node connections.
Positive Edge Percentage	0.879	0.977	+0.098	0.004	**	Proportion of nodes associations that are positive.
Density	0.308	0.150	-0.158	0.068	•	Proportion of possible relationships that are actualized.
Betweenness Centrality Dissimilarity			0.786	0.126		Degree of shift to which nodes make up the shortest paths.
Microbe Dissimilarity	0.843	0.908	+0.065	0.162		Average microbial pair dissimilarity.
Modularity	0.335	0.264	-0.071	0.213		Strength of distinction between clusters.
Node Connectivity	2	0	-2	0.215		Minimum nodes to remove for separation into 2+ components.
Edge Connectivity	2	0	-2	0.235		Minimum edges to remove for separation into 2+ components.
Path Length	1.113	1.328	+0.215	0.367		Average steps in the shortest path between nodes.
Closeness Centrality Dissimilarity			0.692	0.482		Degree of shift in which nodes are closest to all others.
Hub Taxa Dissimilarity			0.600	0.790		Degree of shift to which nodes act as network hubs.
Natural Connectivity	0.163	0.166	+0.002	0.955		Average eigenvalue of all network nodes.
Eigenvector Centrality Dissimilarity			0.381	0.998		Degree of shift to which nodes are most influential.
